# Impacts of Climate, Organic Management, and Degradation Status on Soil Biodiversity in Agroecosystems Worldwide

**DOI:** 10.1111/gcb.70486

**Published:** 2025-09-12

**Authors:** Pablo Sánchez‐Cueto, Martin Hartmann, Laura García‐Velázquez, Beatriz Gozalo, Victoria Ochoa, Giulia Bongiorno, Ron Goede, Melpomeni Zoka, Nikolaos Stathopoulos, Charalampos Kontoes, Luis Daniel Olivares Martinez, Jorge Mataix‐Solera, Fuensanta García‐Orenes, Tomas Van De Sande, Helle Hestbjerg, Ina Alsina, Zoltán Tóth, María Paula Barral, Ximena Sirimarco, Joseph Blaise Dongmo, Julienne Nguefack, Rochana Tangkoonboribun, Anna Clocchiatti, Radu Ghemis, Montse Bosch, Marcos Parras‐Moltó, Cristina Yacoub‐Lopez, Santiago Soliveres, Salvado Lladó

**Affiliations:** ^1^ Leitat Technological Center Applied Microbiology and Biotechnologies Barcelona Spain; ^2^ PhD in Biotechnology, Faculty of Pharmacy and Food Science University of Barcelona Barcelona Spain; ^3^ Department of Environmental Systems Science, Institute of Agricultural Sciences ETH Zurich Zurich Switzerland; ^4^ Multidisciplinary Institute for Environmental Studies “Ramón Margalef”, University of Alicante Alicante Spain; ^5^ University Institute for Research on Olive Groves and Olive Oil – INUO, University of Jaén Jaén Spain; ^6^ Soil Biology Group, Wageningen University & Research Wageningen the Netherlands; ^7^ National Observatory of Athens, Operational Unit “BEYOND Centre for Earth Observation Research and Satellite Remote Sensing” Institute for Astronomy, Astrophysics, Space Applications and Remote Sensing Athens Greece; ^8^ Department of Agrochemistry and Environment, Soil Science and Environmental Technologies Group Miguel Hernandez University Elche Spain; ^9^ Inagro vzw Rumbeke‐Beitem Belgium; ^10^ Danish Technological Institute Taastrup Denmark; ^11^ Latvia University of Life Sciences and Technologies Jelgava Latvia; ^12^ Hungarian University of Agriculture and Life Sciences Institute of Agronomy Keszthely Hungary; ^13^ IPADS EEA Balcarce INTA‐CONICET Balcarce Argentina; ^14^ University of Yaounde I Yaoundé Centre Region Cameroon; ^15^ Thailand Institute of Scientific and Technological Research Mueang Pathum Thani District Thailand; ^16^ University of Amsterdam, Institute of Biodiversity and Ecosystem Dynamics Amsterdam the Netherlands; ^17^ Department of Genetics, Microbiology and Statistics University of Barcelona Barcelona Spain

**Keywords:** DNA metabarcoding, global climate, organic farming, soil biodiversity, soil degradation, soil ecology

## Abstract

Unsustainable soil management, climate change, and land degradation jeopardize soil biodiversity and soil‐mediated ecosystem functions. Although the transition from conventional to organic agriculture has been proposed as a potential solution to alleviate these pressures, there is limited evidence of its effectiveness in enhancing belowground biodiversity across different biogeographical regions, climates, and land degradation levels. In this study, we holistically assessed the status of soil biodiversity, from microorganisms to meso‐ and macrofauna, in agroecosystems distributed across four continents. We identified the primary environmental community composition drivers and assessed the effects of the transition from conventional to organic management (no chemical inputs) on soil ecology. Our findings highlight the mean temperature and precipitation of the warmest and coldest quarters of the year, aridity, pH, and soil texture as the primary drivers of the different soil biodiversity components. Overall, organic farming has a significant but small impact on soil biodiversity compared to the other community drivers. On top of that, the results demonstrate the importance of a regional‐specific context for a future generalized transition towards organic soil management. Specifically, under the most arid conditions in our study, organic management showed potential to buffer biodiversity loss in highly degraded soils, with a significant increase in diversity for prokaryotes and protists compared to conventionally managed soils. Therefore, the combination of a global and, simultaneously, regional‐specific approach supports the hypothesis that a shift towards organic agriculture would maximize its beneficial impact on belowground diversity in highly degraded soils under arid conditions over the coming years, being a crucial tool to increase resilience and adaptation to global change for agriculture.

## Graphical abstract

Unsustainable farming, soil degradation, and climate change threaten the biodiversity of life within soils, which is essential for healthy ecosystems. However, the effects of sustainable farming strategies, such as organic agriculture, on soil biodiversity are still poorly understood. In this work, we assessed soil biodiversity status in agroecosystems across four continents and the impact of soil management on its composition. Our results show a region‐specific positive effect of organic farming on soil biodiversity, especially in degraded soils from highly arid regions. This suggests that wider adoption of organic practices in these disturbed regions could enhance soil resilience to global change.

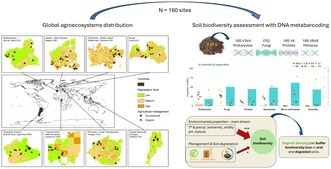

## 1 Introduction

Soil constitutes the fundamental basis of terrestrial ecosystems and is crucial for maintaining the natural contributions and services that sustain human society (Bardgett and van der Putten 2014; Kadykalo et al. 2019; Jansson and Hofmockel 2020). Moreover, soil is considered one of the most important reservoirs of biodiversity on Earth, being estimated to harbor between 25% and 60% of the total biodiversity across the tree of life (Decaëns et al. 2006; Thompson et al. 2017; Anthony et al. 2023). In addition, soil organisms are pivotal regulators of a broad number of processes that underpin global biogeochemical cycles and simultaneously provide multiple ecological functions such as nutrient cycling, carbon storage, water purification, and support for plant productivity (Singh et al. 2010; Wall et al. 2015; Saccá et al. 2017). However, the complexity of life forms below the soil surface (bacteria, archaea, fungi, protozoa, nematodes, earthworms, and other invertebrates), their distribution and relationship with soil properties (texture, carbon content, etc.), and climatic conditions (temperature, precipitation, aridity, etc.) challenge understanding of the current soil biodiversity status (FAO 2020; Aslani et al. 2022; Köninger et al. 2023; Labouyrie et al. 2023). This understanding is crucial for predicting decreases and shifts in diversity, which are essential for designing effective strategies for monitoring, protecting, and conserving soil biodiversity in a global context defined by the increasing importance of multiple soil stressors (Gardi et al. 2009; FAO 2020; Rillig et al. 2023).

Unsustainable soil management practices and soil degradation, together with climate change, are major stressors that can disrupt the delicate balance of soil ecosystems (Smith et al. 2016; Ekka et al. 2023). This is particularly relevant in agroecosystems, where an intensification process has been conducted to reach food demands during the last century, with external chemical inputs at the forefront significantly contributing to land degradation, increased greenhouse gas emissions, lower soil fertility, and global biodiversity loss (Maitima et al. 2009; Gomiero et al. 2011; Smith et al. 2016; Zabel et al. 2019). In response to this, global policy initiatives, such as the Kunming–Montreal Global Biodiversity Framework, promote sustainable biodiversity management in agroecosystems. These efforts aim to enhance the resilience, efficiency, and productivity of agricultural systems, thereby contributing to food security while conserving biodiversity. Following this vision, the urgent maximization of the percentage of agricultural land area covered by sustainable management is encouraged by recent directives, such as the Farm‐to‐Fork strategy in the European Union (EU), which has set an ambitious objective of 25% organic agriculture by 2030. However, the impact of this transition towards more sustainable agriculture on soil biodiversity remains unclear.

Several studies have shown how agricultural organic management can promote the diversity of prokaryotic and eukaryotic soil organisms (Lentendu et al. 2014; Hartmann et al. 2015; Del Duca et al. 2024; Phillips et al. 2024). However, these effects can be modulated or outweighed by other soil biodiversity drivers, such as climate, soil properties, and degradation (Oliver and Morecroft 2014; Lemanceau et al. 2015). In addition, not all soil organisms respond uniformly to soil management, and taxa‐dependent responses to organic farming may be expected (Cozim‐Melges et al. 2024). Thus, the lack of geographically extensive studies comparing the impacts of conventional and organic soil management on soil biodiversity (from microorganisms to macrofauna), while accounting for region‐specific soil and climatic variables, has led to a significant knowledge gap. This is particularly relevant in the context of the Kunming–Montreal Global Biodiversity Framework (CBD 2022) and the EU Soil Monitoring Law (Council of the European Union 2024), both of which call for improved understanding and protection of soil biodiversity in agricultural landscapes.

**FIGURE 1:**
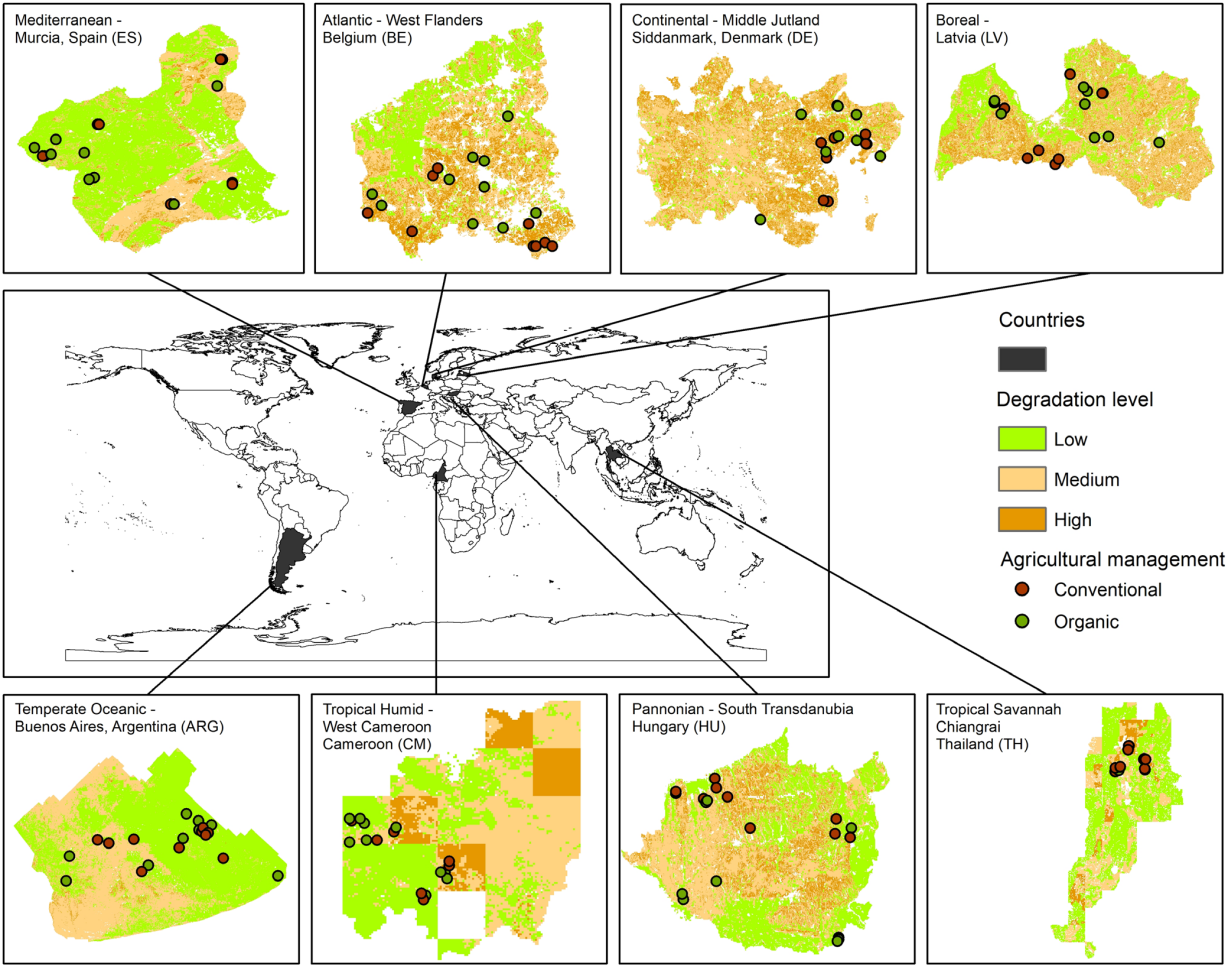
Distribution of the 160 croplands sampled. Both red and dark green points represent the location of conventional and organic agricultural management plots, respectively. These points are situated in areas with varying levels of soil degradation (low, medium, and high), indicated by co‐assessing (within each country) soil erosion and soil organic carbon for each soil texture class. Map lines delineate study areas and do not necessarily depict accepted national boundaries.

To address this critical gap, this study presents an experimental framework encompassing 160 agricultural sites (hereafter also referred to as agroecosystems or croplands) from eight regions located on four different continents, covering multiple biogeographical conditions and different soil degradation levels (Figure 1). In each of the eight regions, the same number of conventional and organic sites were selected. For each site, soil biodiversity was assessed in a harmonized and comparable manner by focusing on six keystone groups of belowground organisms: prokaryotes, fungi, protists, nematodes, micro‐arthropods, and annelids. The diversity of each taxon was assessed using metabarcoding with target‐specific primers. In addition, multiple soil features and climatic variables were obtained for each site along with soil degradation data. Therefore, the primary aims of the current study were (i) to increase our understanding of soil biodiversity status in agroecosystems from different biogeographical regions and the responses to environmental drivers by soil organism type and (ii) to evaluate the impact of soil management strategies (conventional versus organic) and soil degradation on soil biodiversity status on global and regional‐specific scales.

## 2 Material and Methods

### 2.1 Experimental Design

In total, 160 cropland sites were selected from five NUTS‐2 regions and three equivalent administrative regions outside Europe (i.e., in the case of Argentina and Thailand, the administrative region closer to the NUTS‐2 EU category would be Province, and in Cameroon, since 2008, it would be Region), covering different biogeographical contexts: Mediterranean—Murcia (ES), Continental—Middle Jutland/Siddanmark (DE), Pannonian—South Transdanubia (HU), Atlantic (West Flanders (BE)), Boreal—Latvia (LV), Temperate Oceanic—Buenos Aires (ARG), Tropical Humid—West Cameroon (CM), Tropical Savannah—Chiangrai (TH). Working at the regional level within countries allowed the current experimental design to be more robust and reduced intra‐country sample variance, particularly in large countries where multiple and very different climates coexist. Therefore, all soil samples were selected from the same climatic region within each country. Although the results were specific to the selected regions within these countries, for ease of interpretation, throughout this study, the regions are referred to by country name. In each of the eight regions, 20 plots were selected in total for two land management types, organic and conventional agriculture, and contrasted soil degradation levels within each land management type (Figure 1). All sites in the region were actual farms with similar soil types and dominant crops (Table S1a). Although each country had its own definition of conventional/organic management, the different farms shared the application (conventional) or no application (organic) of chemical fertilizers and pesticides within the last 10 years.

### 2.2 Soil Degradation Assessment

Two of the major processes that lead to soil degradation are soil erosion and organic carbon decline (Prăvălie 2021; Wang et al. 2023). Hence, soil erosion (t/ha/year) data from the Revised Universal Soil Loss Equation (RUSLE) 2015 dataset from the European Soil Data Centre—Joint Research Centre (ESDAC—JRC) (Panagos et al. 2015, 2020) and organic carbon data (t/ha) from the Global Soil Organic Carbon Dataset (FAO and ITPS 2018) were combined and co‐assessed with soil texture information (ESDAC—JRC for the EU or ISRIC—World Soil Information for international regions) to classify the degradation status of soils within each region. The spatial resolution of the soil erosion dataset was 100 × 100 m for the European regions, 250 × 250 m for Argentina, and 25 × 25 km for Thailand and Cameroon owing to the unavailability of higher‐resolution data. For the FAO Global Soil Organic Carbon Dataset, the spatial resolution was 1000 × 1000 m for all regions. The different dimensions of resolution between datasets were homogenized by resampling the information of the soil organic carbon dataset to a pixel size of 100 × 100 m for European soil and 250 × 250 m for Argentinian soil, or by resampling the soil erosion information to a pixel size of 1000 × 1000 m for Cameroon and Thailand soils. Using the natural break classification (Jenks) method (Jenks 1967), the soil degradation levels were determined based on inherent groupings within the data variability. These degradation maps provided the basis for the design of soil sampling campaigns. Consequently, the three initial levels of soil degradation (1, low; 2, medium; and 3, high) were merged into two levels (Low = low/medium and High = medium/high) within each region to obtain an adequate number of samples per management for statistical analysis (five sites per condition; Table S1a).

### 2.3 Soil Sampling

Between June and November 2022, all croplands were sampled. The soil sampling protocol used in this study was in accordance with the Land Use/Land Cover Area Frame Survey (LUCAS) (Orgiazzi et al. 2018) and Soil Biodiversity Observation Network (SOILBON) (Guerra et al. 2021) international initiatives. First, a representative point in the field was identified, ensuring that soil type, slope, and management practices were homogeneous within a 1.5‐m radius in all directions from the point (no transitional or waterlogged areas). Five subsamples (one central and four from each cardinal direction) were collected using a 5 cm diameter × 10 cm depth PVC soil corer and pooled in a single bag. Visible roots and stones were carefully removed from all soil samples before sieving them through a 2‐mm mesh and dividing them into two subsamples. Subsequently, one subsample of 100 g was freeze‐dried for DNA extraction, and the second subsample was air‐dried at room temperature (20°C–25°C). Both subsamples were sent to the Department of Ecology at the University of Alicante, where DNA extraction and physicochemical analyses were performed. All the materials used in this study were cleaned between samples with paper wipes and 70% ethanol.

### 2.4 Soil Environmental Variables

Several environmental variables were considered to assess the primary soil biodiversity drivers in global agroecosystems, including physical and chemical soil properties, geographical location, and climate (Table S1b).

The soil properties were measured using various standardized laboratory methods. Briefly, soil pH and electrical conductivity were determined from a 1:5 (soil:water) solution using lab pH and conductivity meters after air‐drying the soil for 1 week and sieving it through a 2‐mm mesh. The total organic carbon (TOC, g kg^−1^) content was analyzed using ^13^C isotopic methods following acid fumigation, thereby avoiding the use of potassium dichromate, in line with EU green‐card standards. Water‐holding capacity (WHC, %) was measured by saturating 20 g of air‐dried soil with 20 mL of deionized water and allowing it to drain for 24 h. Available phosphorus was extracted using 0.5 M NaHCO_3_, followed by centrifugation, and the phosphorus concentration (mg P kg^−1^ soil) was determined using the malachite green method. Ammonium (NH_4_) and nitrate (NO_3_) concentrations (mg P kg^−1^ soil) were determined after extraction with 1 M KCl and subsequent chemical conversion. Soil texture (%), including clay, silt, and sand content, was measured using a slurry of air‐dried soil and a 0.5% sodium hexametaphosphate solution, with sand content determined by sieving and silt and clay content determined by sedimentation. Infiltration was assessed by placing 20 g of soil in a funnel, saturating it with water, and measuring the time required for 50% of the water to pass through. Overall, these variables were considered a good representation of soil physicochemical characteristics derived from soil type and management, which can directly influence the biodiversity status of the soil.

Historical climatic data, including annual, seasonal, and coldest–warmest quarters of precipitation and temperature, were extracted from WorldCLIM 2.0 and aggregated for the 1970 to 2000 period at a 1 km^2^ resolution (Fick and Hijmans 2017). Annual potential evapotranspiration and the aridity index for each sampling site were extracted from Global Aridity and Potential Evapotranspiration (CGIAR‐CSI) datasets, respectively, using the same 1970–2000 period (Zomer et al. 2022).

It should be noted that some of these environmental variables were highly correlated; thus, the selection of specific predictors was performed following stepwise model selection using permutation tests (Blanchet et al. 2008; Section 2.6).

### 2.5 DNA Extraction, Library Preparation, and Bioinformatics

DNA was extracted from 0.25 g of freeze‐dried soils from each plot using the DNeasy PowerSoil Pro HTP 96 Kit (Qiagen Inc., Valencia, CA, USA). Amplicon libraries were generated, targeting the four selected ribosomal gene regions based on literature (Sapkota and Nicolaisen 2015; Frey et al. 2016; Tedersoo and Lindahl 2016; Sikder et al. 2020; Guerra et al. 2021) and specified in Table S1e. Sequencing was performed on the Illumina NextSeq platform PE300 60 M reads following the recommendations of Illumina Inc. Sequencing was performed by Genome Québec Inc. (*Centre d'expertise et de services Génome Québec, Montréal*, Quebec, Canada) pooling libraries based on the expected amplicon length. The sequencing output was similar across all four primers in terms of the number of high‐quality reads (approximately 20 M reads per amplicon, with > 90% of high‐quality reads > Q30). Raw sequencing data were processed using a bioinformatics pipeline largely based on VSEARCH (Rognes et al. 2016) as described by Longepierre et al. (2021). Briefly, primers were trimmed from paired‐end reads using Cutadapt (Martin 2011), then the paired‐end reads were merged using VSEARCH. Quality filtering by maximum expected error was conducted with VSEARCH, and the UNOISE algorithm (minsize 8) was used for delineating sequences into amplicon sequence variants (ASVs); then chimeras were removed (VSEARCH, Edgar 2016) and verified biological targets using Metaxa2 (Bengtsson‐Palme et al. 2015) for the 16S and 18S rRNA genes or ITSx (Bengtsson‐Palme et al. 2013) for ITS2. Subsequently, the taxonomic classification of each verified ASV was performed by running the SINTAX algorithm implemented in VSEARCH against public databases with a minimum bootstrap cutoff of 0.8. The databases used were SILVA v.138 (Pruesse et al. 2007) for the 16S V3V4 region (prokaryotes), the UNITE v.8.3 database (Abarenkov et al. 2010) for the ITS2 region (fungi), and the PR2 v5.0 database combined with SILVA v.138 (including only metazoans) for the 18S V4 (protists) and 18S V6V8 regions (nematodes, micro‐arthropods, and annelids). Metazoan taxonomy levels from the databases were harmonized and adjusted to the classic Linnean levels from NCBI with an R script mostly based on the taxonomizr package v0.11.1 (Sherrill‐Mix 2019) to facilitate subsequent analysis.

### 2.6 Statistical Analysis

Independent datasets were generated for each group: prokaryotes (domain: bacteria and archaea), fungi (kingdom: fungi), protists (clades: TSAR, Amoebozoa, Crums, Cryptista, Excavata, Haptista, Provora, and Chlorophyta), nematodes (phylum: Nematoda), micro‐arthropods (class/order: Collembola, Sarcoptiformes, Trombidiformes, and Mesostigmata), and annelids (phylum: Annelida) and further processed in R software (version 4.1.2). Rarefaction curves were generated for each target using the ranacapa R package (version 0.1.0) (Figure S1) to define the minimum counts per sample. Following the recommendation of Schloss (2024), the ASV tables were 100‐fold iteratively subsampled to 30,352, 7912, 1108, 1268, 156, and 55 reads for prokaryotes (*N* = 160), fungi (*N* = 158), protists (*N* = 153), nematodes (*N* = 159), micro‐arthropods (*N* = 121), and annelids (*N* = 90), respectively. All subsequent statistical analyses were performed independently for individual organisms based on the rarefied ASV count table.

Alpha diversity analysis was performed by calculating the Shannon diversity index using the *diversity* function in the vegan package in R. Beta diversity was assessed based on Bray–Curtis dissimilarity matrix calculation using the *vegdist* function in the vegan package (version 2.6‐4). The rationale for using both the Bray–Curtis and Shannon indices is their ability to capture different aspects of community diversity, being particularly effective for handling sparse data and defining the community structure or providing a comprehensive but simple measure of alpha diversity, respectively. However, given the availability of alternative biodiversity indices and normalization methods, Spearman correlations were performed between different indices—observed richness versus Shannon diversity or Bray–Curtis versus Jaccard dissimilarity—as well as between different normalization approaches—Scaling with Ranked Subsampling (SRS, Beule and Karlovsky 2020) versus rarefaction (Schloss 2024)—to assess comparability with other valid approaches. The strong correlations obtained (rho = 0.8–1; *p* < 0.001) suggest that the choice of the diversity metrics and normalization method did not substantially alter the results. Shannon and Bray–Curtis ecological metrics, combined with taxonomical abundances at different resolution levels (phylum, genus, or ASVs), were explored to establish the soil biodiversity status in agroecosystems (differences and similarities across all regions) and assess the management effect dependent on historic climate, soil properties, and degradation.

First, to identify biodiversity differences between regions, nonparametric tests were performed. The Kruskal–Wallis test with the post hoc Dunn test (adjusted using the Benjamini–Hochberg correction) for alpha diversity and pairwise permutational analysis of variance (PERMANOVA) (pairwise.adonis; Martinez Arbizu 2020) for beta diversity differences were applied. In addition, Levene's test (*leveneTest* function, car package Version 3.1‐3) for alpha diversity and permutational analysis of multivariate dispersions (PERMDISP) (*betadisper* function, vegan package) for beta diversity were applied to test for homogeneity of variance between factor levels. Subsequently, to identify key environmental drivers of these metrics and define an “optimal” model, a forward selection of all the environmental variables measured under this study (explained in the previous section) was applied using the *OrdiR2step* function from vegan (Oksanen et al. 2020). To maximize the explanatory power and avoid overfitting, the following stopping criteria were applied for variable selection: adjusted *R* ^2^ decreased, exceeded the full model adjusted *R* ^2^ or when the significance is exceeded (*p* < 0.05) after 999 permutations (Blanchet et al. 2008, output in Table S2). To evaluate both the importance and the effect trends of the environmental drivers on biodiversity, Spearman's rank correlations (psych package, version 2.4.12) on alpha diversity and distance‐based redundancy analysis (dbRDA) (vegan package) on beta diversity were performed. In addition, correlations between taxa relative abundance at different levels (phylum, order, or genera) and the ordination scores were determined using the *envfit* function (vegan package) with 999 permutations. Regarding biodiversity similarities between agroecosystems, a combination of the UpSetR (version 1.4.0) and ComplexUpset packages (version 1.3.3) was used to identify and represent shared or “core” versus “unique” ASVs across regions (minimum occurrence in one sample per region).

To assess the significant impacts of management, degradation, and their interactions with environmental variables, PERMANOVAs with 999 permutations were performed on alpha diversity, beta diversity, and ASV relative abundance. This approach was consistently replicated at (1) global (all sites together) and (2) regional‐specific levels (only sites within the same region). Thus, two types of models were tested for each organism‐related metric (individual model details in Table S3):

1. biodiversity~management * degradation * (climate + soil properties + location)
2. biodiversity~management * degradation + environmental covariate

Environmental factors included in each model were selected based on prior significant results from *OrdiR2step* (Table S2). This pre‐selection, along with the exclusion of interactions among environmental variables, was done to avoid overparameterization and improve interpretation of the models, which aimed to assess the context dependency of management impacts on biodiversity. *p*‐values of 0.05 were established as significant for alpha and beta diversities, whereas adjusting for multiple testing and controlling for false discovery rate (Benjamini and Hochberg 1995) was applied for ASV analysis, using the *p.adjust* function from the stats package (Version 4.4.2) and considering a *q* value of < 0.1 as statistically significant (< 10% chance of being a false positive). Before conducting the management‐sensitive ASV analysis, rare ASVs were removed within each region and organism group (defined as < 0.5% total relative abundance and occurring only in one sample). Significant ASVs were *z*‐transformed using the R *scale* function to visualize relative abundance differences between conventional and organic sites across regions.

A summary diagram of the main experimental design, including the methods used to assess soil biodiversity and the statistical analysis workflow with their respective objectives, is shown in Figure S2.

## 3 Results

### 3.1 Soil Biodiversity Status in Agroecosystems and Links With Environmental Variables

This study captured a broad range of biodiversity within the soil from agricultural sites across four continents, with a clear regional influence on taxa distribution. In total, 57,718 ASVs were identified from prokaryotes, 15,816 from fungi, 12,900 from protists, 2304 from nematodes, 1453 from micro‐arthropods, and 120 from annelids. Furthermore, 90 phyla, 262 classes, 682 orders, 1316 families, and 2540 genera were overall successfully annotated. Within each organism group, most of the soils studied presented a consistently high dominance of *Actinobacteriota* (prokaryotic phylum, 31% mean relative abundance), *Ascomycota* (fungal phylum, 83% mean relative abundance), *Cercozoa* (protist phylum, 70% mean relative abundance), *Rhabditida* (nematode order, 63% mean relative abundance), *Sarcoptiformes* (micro‐arthropod order, 25% mean relative abundance), and *Crassiclitellata* (annelid order, 39% mean relative abundance) (Figure S3). The eight regions studied shared 2171 (~4%) prokaryotic ASVs, 114 (~1%) fungal ASVs, 89 (~1%) protist ASVs, 24 (~1%) nematode ASVs, and zero micro‐arthropod/annelid ASVs (Figure S4a). Among these identified as “core ASVs,” several of the most prevalent and abundant belonged to the genera *Bacillus*, *KD4‐96, Bradyrhizobium*, *Streptomyces*, and *Blastoccoccus* for prokaryotes; *Nectiriaceae, Fusarium, Sordariomyces, Cladosporium, Chaetomiaceae*, *and Penicillium* for fungi; *group_T, Sandonide* (family), *Oxytrichidae* (family), and *Leptophyridae* (family) for protists; and *Cephalobus, Merlinius*, and *Plectus* for nematodes (Figure S4b). However, the high percentage of regional‐specific or “unique” ASVs (prokaryotes: 25%, fungi: 50%, protists: 45%, nematodes: 49%, micro‐arthropods: 64%, and annelids: 43%) indicates the unique biodiversity harbored by soils depending on their origin (Figure S4a). This was particularly true for soils from Thailand, Cameroon, and Spain, which exhibited the highest cumulative relative abundances of unique ASVs across the different organisms studied (Figure S4a) and a small number of shared ASVs with the other regions (Figure S4b).

**FIGURE 2:**
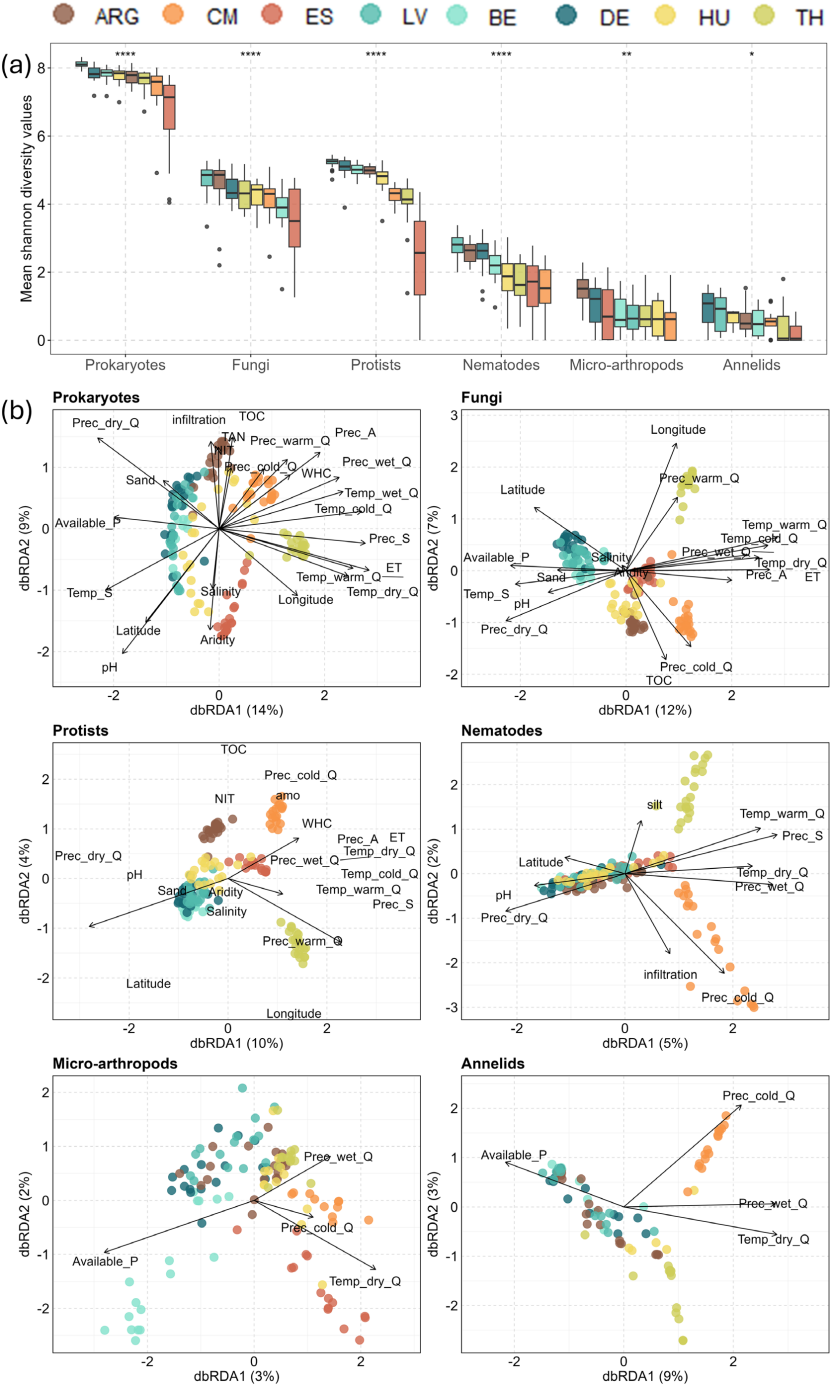
Biogeographical patterns and environmental properties influence soil biodiversity. (a) Shannon index values (alpha diversity) distribution across the eight regions studied. The Shannon index was calculated for each sample based on the observed ASVs and their evenness for prokaryotes, fungi, protists, nematodes, micro‐arthropods, and annelids. The distribution by region and organism is represented as a boxplot, and the mean values are indicated as a horizontal bar, colored by region. The significant impact of the sample origin (region) on alpha diversity was calculated with the Kruskal–Wallis test, and asterisks were added when significant (*p < 0.05, **p < 0.01, ****p < 0.0001). (b) dbRDA analysis, by organism group, on the Bray–Curtis dissimilarity matrix calculated from ASV counts, showing the influence of soil physicochemical properties*^1^, bioclimatic variables collected from 1970 to 2000*^2^ and location*^3^ on global beta diversity samples ordination. The samples were color‐coded based on region of precedence. Prior to the analysis, the relevant variables were selected by each group with ordiR2step analysis (Table S2a). The region name is abbreviated and color‐coded by the country of origin: ARG‐Argentina, DE‐Denmark, HU‐Hungary, LV‐Latvia, Be‐Belgium, ES‐Spain, TH‐Thailand, CM‐Cameroon. *1 total organic carbon (TOC), total available nitrogen (TAN), pH, phosphate availability (Available_P), salinity, water‐holding capacity (WHC), clay, sand and silt content, nitrate available (NIT), ammonium available (AMO). *2 annual, seasonal and coldest‐warmest‐wettest quarters of precipitation (Prec_A, Prec_S, Prec_cold_Q—Prec_warm_Q‐Prec_wet_Q) and temperature (Temp_A, Temp_S, Temp_cold_Q—Temp_warm_Q‐Temp_wet_Q), aridity and potential evapotranspiration (ET0). *3 latitude and longitude.

The regional effects on soil biodiversity were further confirmed at the alpha (Kruskal–Wallis < 0.05) and beta diversity levels (pairwise PERMANOVA *p* < 0.05) (Figure 2; Tables S4a and S5a). However, the significant differences in terms of dispersion homogeneities within regions (Levene and PERMDISP tests *p* < 0.05; Tables S4b and S5b) indicate that these differences are partially driven by variance heterogeneities. The Shannon index (alpha diversity) values revealed that soils from Latvia (LV), Argentina (ARG), and Denmark (DE) consistently harbored the most diverse soil communities, particularly for prokaryotes (range of mean values between regions: 7.7–8.1), fungi (4.4–4.7), protists (5–5.2), and nematodes (2.5–2.8) (Figure 2a). These alpha diversity values were statistically different from those observed in soils from Spain (ES) (*dunnTest p*.adj < 0.05; Table S4a) and were overall significantly negatively impacted by high temperatures, such as the mean values recorded during the driest quarter of the year (protists: *R* ^2^ = 42%, rho = −0.71; nematodes: *R* ^2^ = 30%, rho = −0.49; prokaryotes: *R* ^2^ = 25%, rho = −0.62; fungi: *R* ^2^ = 9%, rho = −0.3) (Table S2a; Table S6). These Spanish soils were characterized by having one of the highest temperatures during the driest quarter of the year and exhibited high clay content and aridity compared to soils from the other regions (Table S1c,d), which were also negatively correlated with the alpha diversities of protists and prokaryotes (Table S6).

For Bray–Curtis dissimilarities (beta diversity), unique and shared environmental drivers of community composition were found for each soil organism (dbRDA; Figure 2b). Soil properties, location, and climate explained varied proportions of compositional variation by organism (prokaryotes: 45%; fungi: 33%; protists: 22%; annelids: 12%; nematodes: 10%; and micro‐arthropods: 7%) (Table S2a). Climatic drivers, particularly temperature and precipitation in extreme year quarters (driest, wettest, coldest and warmest), strongly influenced beta diversity, with samples from Thailand and Cameroon clustering separately from those from Europe and Argentina. TOC was the key to differentiating microbial communities in Argentina and Cameroon soils. Aridity, salinity, phosphorus, and pH were the most important drivers of the ordination of European soil samples (Figure 2b). For example, aridity was particularly relevant for the clustering of prokaryotic communities in Spanish and Hungarian soils (Figure 2b). The same dbRDA results also showed specific taxa within each type of soil organism with significant weight in biodiversity ordering (Figure S5). Briefly, Thai soils showed a higher abundance of the prokaryotic phyla *Euryarchaeota*, *Crenarchaeota*, and *Halobacterota* than samples from other countries, which was positively linked to precipitation seasonality, evapotranspiration, and temperature of the driest quarter. *Chloroflexi* and *Planctomycetota* were important for Spanish and Thai ordination, and their abundances were positively correlated with mean temperatures in the warmest and driest quarters of the year. For other soil organisms, Thai clustering was defined as *Entorrhizomycota* (fungi), *Chlorophyta* (protists), and *Tubificida* (annelids). The abundance of these soil organisms was positively correlated with mean precipitation in the warmest quarter of the year (except for annelids). European soils were enriched in *Bacteroidota* (prokaryotes), *Mortierellomycota* (fungi), *Basidiobolomycota* (fungi), and *Crassiclitellata* (annelids), with available phosphorus as a key influence. Concurrently, *Sarcoptiformes* (micro‐arthropods) were associated with Cameroon and Spanish soils and were influenced by temperature in the driest quarter of the year. At higher taxonomic resolution, specifically for fungi, several genera from *Entorrhizales* and *Trichosphaeriales* were positively associated with precipitation in the warmest quarters, while *Fusarium* and *Staphylotrichum* were associated with precipitation in the coldest quarters and elevated TOC content (Figures S6 and S7).

### 3.2 Impacts of Soil Management Strategies and Soil Degradation on Soil Biodiversity Status

**FIGURE 3:**
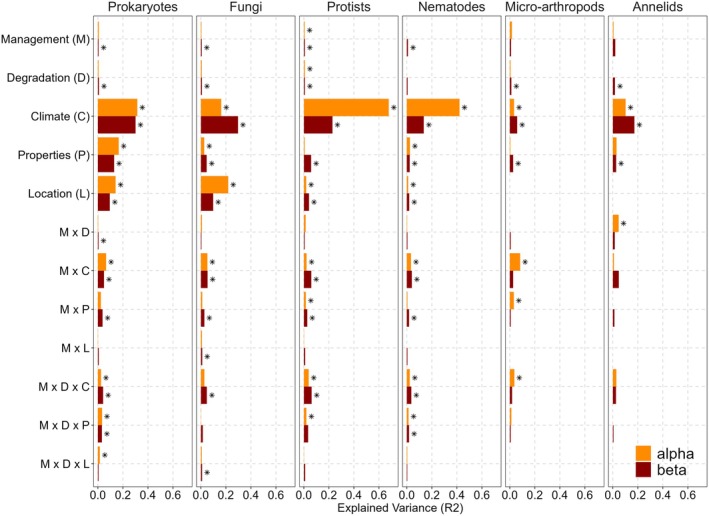
Global impacts of main factors on soil biodiversity. These included first management strategies (M, conventional vs. organic) and degradation levels (D, low vs. high). Then, climate variables (C, average bioclimatic data from 1970 to 2000—temperature, precipitation, aridity, and evapotranspiration‐related variables), soil properties (P, physicochemical data collected from soils), and location (L, latitude and longitude) were included alone or in combination with M and D to see the context dependency of the main factors' effects. Explained variance (*R* ^2^) and significance were calculated using PERMANOVA (permutations = 999) and are displayed as bar plots color‐coded by diversity metric (alpha Shannon = yellow; beta Bray–Curtis: Red). Asterisks indicate significant effects (*p < 0.05). Note that C, P, and L each comprise multiple variables that were assessed independently in the same model (Table S7a,b); in the figure, bars represent the cumulative *R* ^2^ of all variables within a category, and asterisks denote significance of at least one variable or interaction in that category.

The first global analysis (all sites combined) explored the impacts of soil management strategies (conventional versus organic), alone or in combination with soil degradation (D) and other environmental variables (climatic variables (C), location (L), and soil properties (P)). This analysis revealed significant and organism‐dependent effects of management strategies on both soil alpha and beta diversity (Figure 3; Table S7b), which were stronger when interactions with other environmental variables (D, C, L, and P) were considered. This was particularly true for prokaryotes, protists, and nematodes (Figure 3; Table S7b). In addition, not all environmental variables related to climate and soil properties contributed equally to the explained variance (*R* ^2^) of the biodiversity (Table S7a), as also shown by the previous dbRDA analysis (Figure 2; Table S2).

Overall, the soil management type showed a significant effect on alpha diversity observed only for protists (PERMANOVA *R* ^2^ = 0.6%, *p* < 0.05; Figure 3; Table S7b). However, several significant patterns emerged when considering the interactions between management and other environmental variables. The most relevant interactions were observed between management and climate‐related variables for all organisms except annelids, with up to 8.2% of the variance in the alpha diversity of micro‐arthropods, 6.6% for prokaryotes, and 5.2% for fungi (Figure 3; Table S7b). In addition, protists were the most sensitive group to management impacts on alpha diversity, dependent on soil degradation and climate (PERMANOVA *R* ^2^ = 3.9%, *p* < 0.05; Figure 3; Table S7b), followed by micro‐arthropods (PERMANOVA *R* ^2^ = 3.6%, *p* < 0.05; Figure 3; Table S7b), prokaryotes (PERMANOVA *R* ^2^ = 2.5% *p* < 0.05; Figure 3; Table S7b), and nematodes (PERMANOVA *R* ^2^ = 2.4% *p* < 0.05). Moreover, a significant three‐way interaction between management, degradation, and soil properties was found for prokaryotes, protists, and nematodes (PERMANOVA *R* ^2^ = 1.4%–3.3% *p* < 0.05; Figure 3; Table S7b).

At the beta diversity level, management effects were statistically more robust than for alpha diversity for prokaryotes, fungi, protists, and nematodes, although they still had a small effect on global compositional variance (PERMANOVA *R* ^2^ = 0.5%–1%, *p* < 0.05; Figure 3; Table S7b). Several interactions were found with climate, soil properties, and degradation level, where the most substantial interactions were seen between management and climate for prokaryotes, fungi, protists, and nematodes (PERMANOVA *R* ^2^ = 4.9%–5.9%, *p* < 0.05; Figure 3; Table S7b). Furthermore, management effects in relation to degradation and climate were observed for prokaryotes, protists, and nematodes, with an explained variance ranging from 3.6% to 6.4% (Figure 3; Table S7b). Similar to alpha diversity, variables related to biogeography (C, L, and P) explained a higher percentage of the variance in the system compared to management and degradation factors.

**FIGURE 4:**
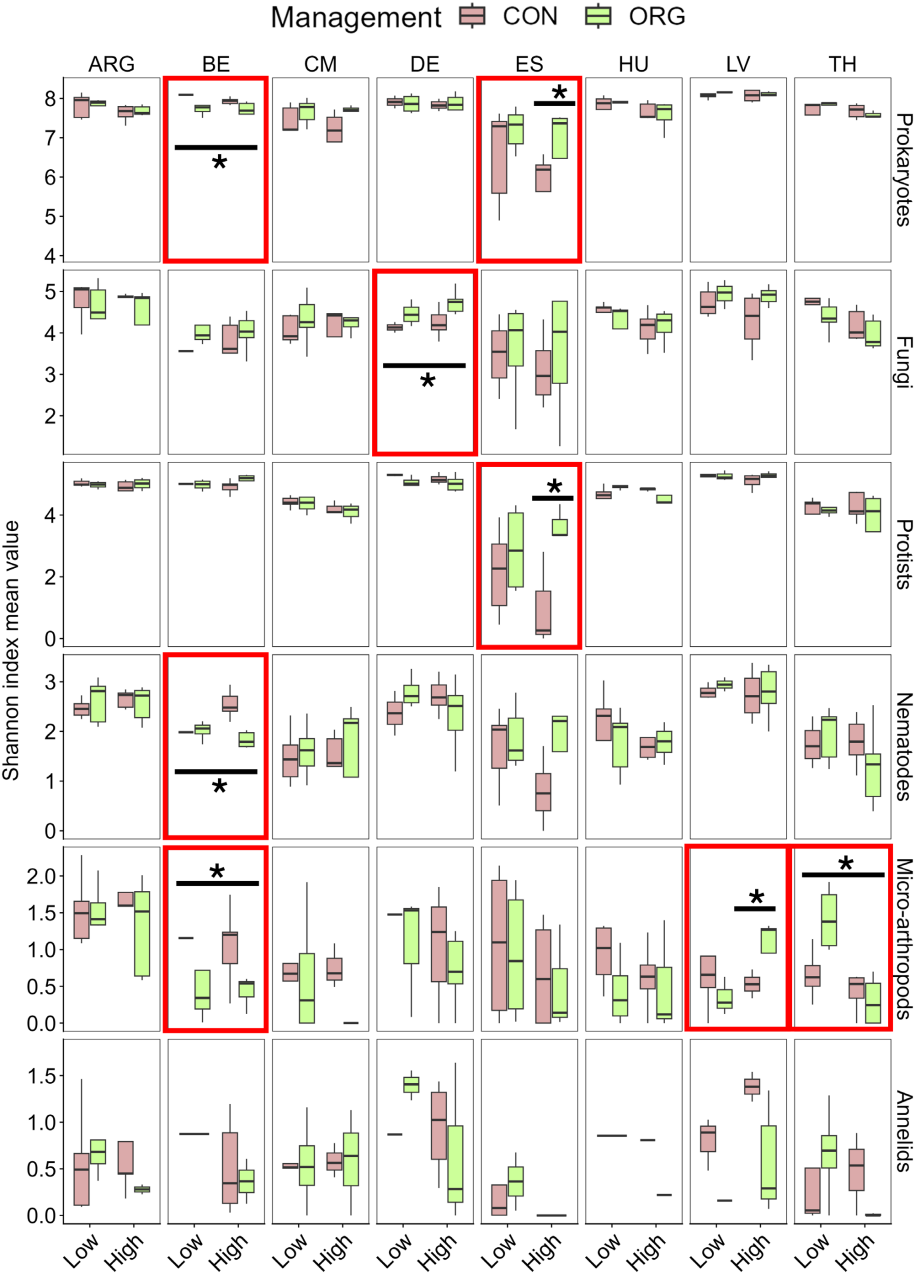
Alpha diversity distribution by management and degradation at the region‐ and organism‐specific level. The distribution of Shannon indices (alpha diversity) by soil organism, region, management (M), and degradation level (D) is represented as boxplots. The mean Shannon diversity values are displayed for each organism and region with a horizontal bar, grouped by Management × Degradation (M×D). From left to right, the countries associated with the regions are displayed: Argentina (ARG), Belgium (BE), Cameroon (CM), Denmark (DE), Spain (ES), Hungary (HU), Latvia (LV), and Thailand (TH). From top to bottom, the organisms are displayed as prokaryotes, fungi, protists, nematodes, micro‐arthropods, and annelids. Boxplots are color‐coded by management type (Conventional—CON: Brown; Organic—ORG: Green), and statistical differences between both are indicated by an asterisk (PERMANOVA M or M×D *p < 0.05; Table S8). Red boxes indicate organism‐ and region‐specific significant effects of management or management × degradation. The long line indicates a significant management effect, while the short line indicates significance at only one degradation level.

Regional‐specific analyses were performed to better define the effects of soil management on soil biodiversity parameters. Individual PERMANOVA confirmed Belgium, Denmark, Spain, and Thailand as the regions most sensitive to management impacts on the alpha diversity of prokaryotes, fungi, nematodes, and micro‐arthropods (PERMANOVA *p* < 0.05; Table S8). In Belgium, organic management led to a consistent reduction in the mean alpha diversity of prokaryotes, nematodes, and micro‐arthropods compared to conventional (Figure 4). Conversely, organic soils from Denmark, Spain, and Thailand showed significant increases in alpha diversity for fungi, protists, and micro‐arthropods, respectively (Figure 4).

Regarding beta diversity, the soil community composition for prokaryotes, fungi, protists, and nematodes from Belgium and Latvia was significantly affected by the type of soil management (PERMANOVA *p* < 0.05; Table S8). In addition, the fungal community composition showed consistent management effects across countries (PERMANOVA *p* < 0.05), except for Thailand (PERMANOVA *p* > 0.1) and Spain (PERMANOVA *p* < 0.1) (Table S8).

**FIGURE 5:**
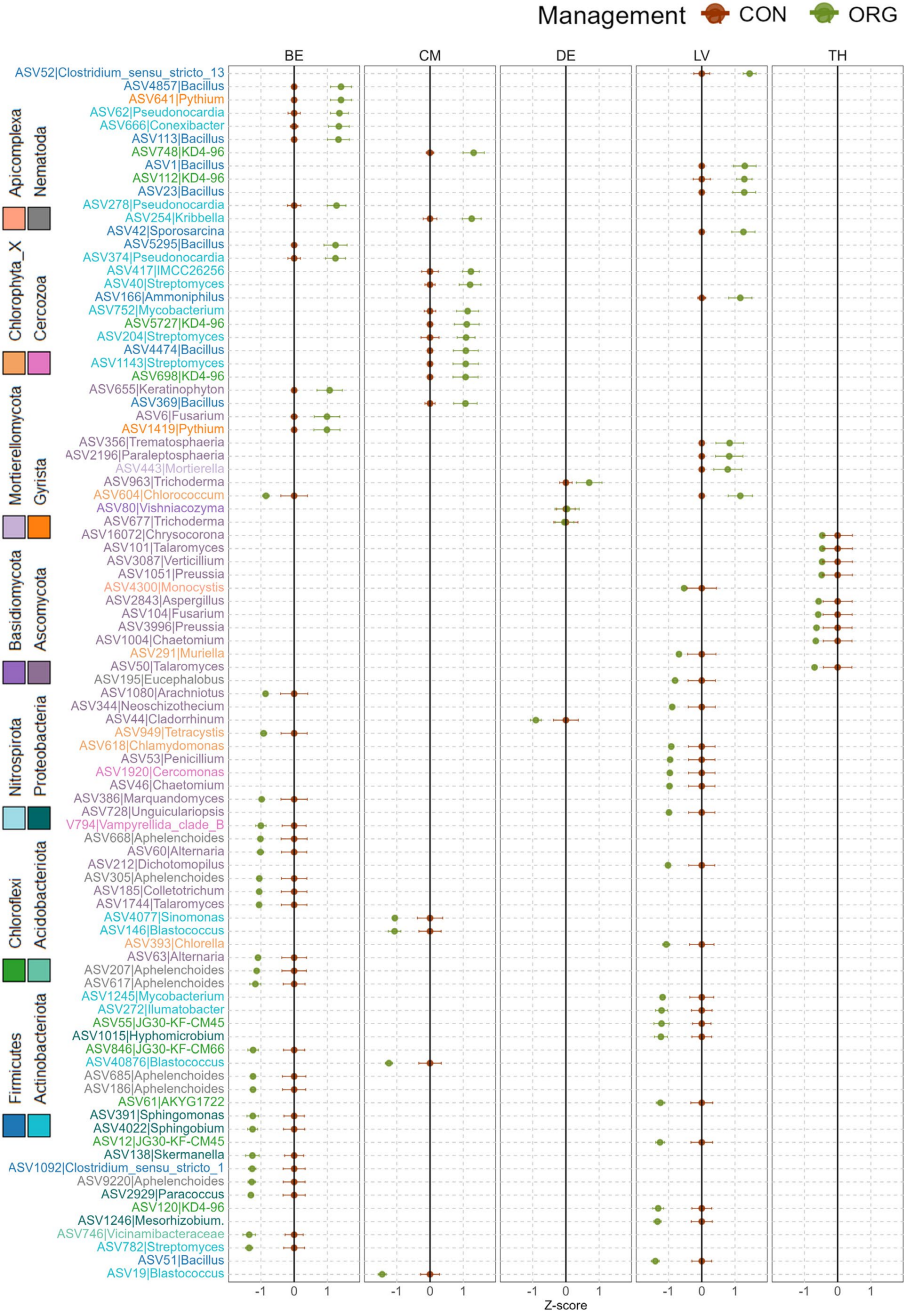
Analysis of management‐sensitive ASVs at a region‐specific level. Representation of the mean *z*‐standardized relative change in abundance between conventional and organic sites of the significant ASVs (PERMANOVA *q* < 0.1) that have been identified in the regions of Belgium (BE), Cameroon (CM), Latvia (LV), Denmark (DE), and Thailand (TH) for prokaryotes, fungi, protists, and nematodes. The brown dots represent the ASV abundance in conventional sites normalized to themselves (conventional–conventional = 0), while the green dots represent the organic abundance normalized to conventional (organic—conventional). The error bars indicate the standard deviation (SE). Positive values indicate an increase in abundance under organic conditions compared to conventional, while negative values indicate a decrease. ASV IDs and the associated genera are shown in the *y*‐axis, color‐coded by their respective phyla. A range of colors was applied based on the higher levels of taxonomy: blue‐green: prokaryotes; purple: fungi; orange‐pink: protists; gray: nematodes.

The regional impact of soil management on soil biodiversity was further confirmed by assessing the management‐sensitive ASVs. In this context, prokaryotes (LV = 194, BE = 130, and CM = 80), fungi (LV = 19, BE = 12, and DE = 11), protists (LV = 19 and BE = 7), and nematodes (BE = 10 and LV = 2) included several ASVs that were either enriched or depleted in organically managed soils compared to conventionally managed soils (PERMANOVA *q* < 0.1; Figure 5; Table S9). By region, among the most sensitive prokaryotes (based on standardized relative changes in abundance), significant increases in ASV relative abundances under organic management were observed for *Bacillus* (BE, LV, and CM), *Streptomyces* (CM), *Mycobacterium* (CM and LV), *Pseudonocardia* (BE and CM), and *Clostridium sensu stricto 13* (LV) (Figure 5). However, in Latvia, different ASVs within the same genus, such as *Bacillus* and *Mycobacterium*, exhibited opposite trends. In addition, ASVs from *Nitrospira* (LV), *Mesorhizobium* (LV), *Blastococcus* (CM), and *Sphingomonas* (BE) were negatively associated with organic management (PERMANOVA *q* < 0.1; Figure 5). Regarding eukaryotes (fungi, protists, and nematodes), ASVs from *Fusarium* (BE, fungi), *Keratinophyton* (BE, fungi), *Trichoderma* (DE, fungi), *Mortierella* (LV, fungi), and *Pythium* (BE, protists) were positively associated with organic management (PERMANOVA *q* < 0.1; Figure 5). Conversely, ASVs from *Fusarium* (TH, fungi), *Penicillium* (LV, fungi), *Alternaria* (BE, fungi), *Tetrascystis* (BE, protists), and *Aphelenchoides* (BE, nematodes) were negatively associated with organic management (PERMANOVA *q* < 0.1; Figure 5).

Finally, the differential impacts of soil management on biodiversity by the degradation status of the soils were investigated. Consequently, when the type of management (M) and soil degradation level (D) were assessed as constraint factors per region, a consistently significant effect on the alpha diversity of prokaryotes and protists in Spanish soils was observed (PERMANOVA *p* < 0.05 *F* = 6; Table S8). The impact of management was stronger in highly degraded soils, where organic management led to a significant increase in the alpha diversity of prokaryotes and protists compared with conventional soils (Figure 4). In addition, a significant management effect dependent on degradation was observed for fungal beta diversity in Spain and Hungary (PERMANOVA *p* < 0.05; Table S8).

Although no significant differences in management and soil degradation interactions were found at the ASV level across any of the eight regions studied (PERMANOVA *q* > 0.1, data not shown), previous observations at the alpha diversity level for highly degraded soils were supported at the ASV level through presence/absence analysis. Specifically, the results showed that 3025 prokaryote, 285 fungal, 56 protist, and 51 nematode ASVs were detected in all Spanish soil conditions, except those with conventional management under high degradation levels (Figure S8).

## 4 Discussion

The current study, using an extensive network of conventional and organic agricultural sites across eight international regions, and holistically assessing soil biodiversity, from bacteria to annelids, revealed: (i) microbial organisms are more widespread than soil fauna, and their distribution is heavily impacted by historically climate‐dependent variables (aridity, temperature and precipitation) and soil properties (pH and nutrient content); (ii) organic soil management significantly shifts soil biodiversity; however, these shifts are regional‐specific and their magnitude is dependent on other environmental variables; and (iii) a transition towards organic management may be more beneficial for soil biodiversity in highly degraded soils from regions suffering multiple stressors, such as unsustainable soil management and aridification.

The primary objective of this study was to increase our understanding of the status of soil biodiversity in agroecosystems. Consequently, the unique and shared components of soil biodiversity across regions located on four different continents were explored. The results showed that a high proportion of core ASVs (ASVs shared in the eight regions studied) belonged to prokaryotes and fungi. In contrast, ASVs belonging to micro‐arthropods and annelids were rare among the core ASVs. This greater diversity of microbes, compared to the more regional‐specific meso‐ and macrofauna, aligns with other studies that assessed some of the soil organisms that were addressed holistically in this study (Bardgett and van der Putten 2014; Delgado‐Baquerizo et al. 2018; Egidi et al. 2019; van den Hoogen et al. 2019; Köninger et al. 2023; Labouyrie et al. 2023). Hence, it is hypothesized that smaller and more diverse groups exhibit greater adaptability and potential for dispersion globally, although to some extent limited, as different shared taxa depend on regional factors. This challenges the paradigm of “everything is everywhere,” as suggested by other studies (Green et al. 2004; Green and Bohannan 2006; Rout and Callaway 2012), and indicates that biogeographical differences influence not only distribution but also the presence or absence of certain taxa, with potential unique contributions to ecosystem functioning (Noronha et al. 2017).

Focusing on the microbial components of the core ASVs (present at least in one site per region), several taxa are considered plant growth‐promoting and stress‐resistant microbes, such as *Bacillus*, *Sphingomonas, Blastococcus*, *Bradyrhizobium*, *Xanthobacteriaceae* (family), *Micrococcaceae*, *Streptomyces*, and *Penicillium* (Li et al. 2023). However, these dominant ASVs also include potential fungal phytopathogens such as the genus *Fusarium* or the family *Nectriaceae* (Lombard et al. 2015), as well as *Cladosporium*, a genus with dual roles as a plant biostimulant due to gibberellin production (Răut et al. 2021) and as an opportunistic human pathogen (Batra et al. 2019). This component of the study aligned with prior efforts to develop a global microbial biodiversity atlas and identify dominant soil members (Delgado‐Baquerizo et al. 2018; Egidi et al. 2019; van den Hoogen et al. 2019), but was more targeted to agroecosystems. Thus, a preliminary step is shown towards defining a core set of “cropland specialist” taxa, which may possess adaptive traits that enable them to thrive across varied climates, soil managements, and/or conditions. In addition, these findings underscore the importance of microbial community surveillance in agroecosystems, considering their dual potential as sources of beneficial species and reservoirs of pathogens with significant contributions to the global One Health concept (Banerjee and van der Heijden 2023).

Moving beyond taxonomy and focusing on diversity metrics, the highest alpha diversity values were observed in Argentina, Latvia, and Denmark. By contrast, alpha diversity results consistently showed significantly lower values across soil organisms in the Spanish soils (characterized by a Mediterranean climate). Moreover, previous negative associations between aridity and microbial biodiversity across various land uses (Maestre et al. 2015; Siles et al. 2023) are aligned with the negative association between aridity and alpha diversity observed in this study, along with low TOC, high clay content, low precipitation, and high temperatures in extreme quarters. Therefore, the present results support previous studies focused on the Mediterranean regions that call for strong protection and restoration of soil biodiversity in agroecosystems under arid conditions (Zdruli et al. 2010; Maestre et al. 2015).

In relation to beta‐diversity analysis, our results also showed that the soil community composition of larger organisms (nematodes, micro‐arthropods, and annelids) was less driven by the same climatic and soil property variables than microbial communities (prokaryotes, fungi, and protists). This variable degree of explanatory power by environmental variables across different groups of organisms is aligned with previous studies across different European biomes that used similar molecular methods (Aslani et al. 2022; Köninger et al. 2023; Labouyrie et al. 2023) and may be attributed to a high dependency of metazoan groups on other non‐measured ecosystem characteristics (feeding interactions, tillage, soil history) (Sánchez‐Moreno et al. 2009; Melakeberhan et al. 2025). The low explanatory power by environmental properties may also reflect the current lack of a well‐established framework for soil fauna assessment using molecular methods, including DNA extraction protocols, primer specificity, and reference databases. Addressing these methodological gaps will be essential for improving holistic soil biodiversity assessments in the near future.

The defined environmental variables were assessed based on the distribution of different components of soil biodiversity across regions. The mean temperature and precipitation of the warmest and coldest quarters, as well as aridity from the historical data collection (1970–2000), followed by in situ measurements of pH, TOC, and soil texture, were considered the key drivers of soil microbial community composition. This aligns with established knowledge, indicating that pH and climatic variables are crucial for soil microbial distribution on a global scale, as observed for bacteria and fungi (Větrovský et al. 2019; Labouyrie et al. 2023). For example, the abundance of the prokaryotic phylum *Chloroflexi* was linked to soil aridity in Spain and to high temperatures in the warmest and driest quarters of the year in soils from Thailand. This is consistent with previous studies on soil warming and aridity, albeit conducted for other soil ecosystems, such as drylands or forests (Maestre et al. 2015; Xie et al. 2019), or not covering different biogeographical regions (Xie et al. 2019; Qiao et al. 2024). Thus, given the ongoing desertification and global warming of the planet (López‐Bermúdez and García‐Gómez 2006; IPCC 2019), soil taxa better adapted to these new conditions may be expected to increase their abundance in agricultural soils from regions affected by environmental stressors closely linked to climate change.

Therefore, regarding soil biodiversity status in agroecosystems, the current results highlight that biogeography drives the taxa distribution. At this precise moment in history, when the global environmental crisis is being exacerbated (climate change and biodiversity loss) (O'Connor et al. 2020), the observation that aridity is correlated with the loss of specific members of soil diversity in agroecosystems indicates that climate change can jeopardize belowground life and the functions that these organisms provide to the ecosystem, and hence, the soil health (Lehmann et al. 2020) and its capacity to produce food in the future. Thus, the momentum that sustainable agriculture has gained in recent years (Montanarella and Panagos 2021; Panagos et al. 2022) should be accompanied by new information about whether these soil management strategies have a beneficial impact on soil biodiversity across biogeographical regions, particularly in those heavily affected by multiple environmental stressors.

In this regard, the current study provides a comprehensive assessment of the impacts of conventional and organic agriculture on multiple soil organisms. The findings revealed that, whereas the global impact of management type (organic versus conventional) on alpha and beta diversities across agroecosystems may be significant for some soil organisms, it was generally minimal. However, significant and stronger interactions emerged when the effects of soil management on soil organisms were assessed in combination with other environmental variables. Although previous studies on smaller scales have confirmed the strong role of soil management strategies in shaping soil biodiversity (Lentendu et al. 2014; Hartmann et al. 2015; Del Duca et al. 2024), our results highlight that the effects of organic management on soil biodiversity varied across biogeographical factors and soil properties. The improvement in predicting bacterial and fungal diversity through a three‐way assessment of soil pH, climate, and land use, as reported by Labouyrie et al. (2024), reinforces the present findings. Thus, by analyzing different soil organisms from four continents and accounting for diverse environmental conditions at each site, the present study expands on previous soil biodiversity research that focused on a limited range of organisms and was confined to regional comparisons (Lentendu et al. 2014; Hartmann et al. 2015; Del Duca et al. 2024), based on meta‐analyses (Cozim‐Melges et al. 2024; Phillips et al. 2024) or including a limited range of variables (Labouyrie et al. 2024).

The Mediterranean soils from Spain, the most arid and vulnerable from a biodiversity perspective investigated in this study, showed a significant increase in the alpha diversity of prokaryotes and protists under organic management, groups previously reported to be negatively affected by arid conditions (Maestre et al. 2015; Chen et al. 2022). Conversely, Atlantic soils in Belgium showed a reduction in alpha diversity for prokaryotes, nematodes, and micro‐arthropods in organic soils. Apart from their specific climatic conditions, these systems were the only croplands based on potatoes. Organic fields in these systems follow different guidelines and pest regulation techniques (Tscharntke et al. 2021) than the cereal‐based fields in the current study, which could explain the differences in terms of alpha diversity shifts. These findings highlight the importance of region‐ and field‐specific management decisions in shaping biodiversity outcomes, with no universal solution. Future experimental validation will be essential to better understand and raise awareness of region‐specific effects that may contribute to broader, even global, biodiversity loss (Gonthier et al. 2014).

Furthermore, the effects of soil management on soil organisms reinforce previous findings from meta‐analyses of the associations between individual soil management practices and shifts in specific taxonomic groups (Cozim‐Melges et al. 2024; Phillips et al. 2024). In this study, prokaryotes (regions affected: CM, ES, LV, BE, and DE), protists (ARG, CM, TH, ES, LV, BE, and DE), fungi (ARG, CM, ES, LV, BE, HU, and DE), and nematodes (DE, BE, and LV) were the soil groups most affected by soil management, which was consistent across the indicated regions. Although the sensitivity of these groups of organisms to soil management strategies has been previously discussed in the literature (Hartmann et al. 2015; Quist et al. 2016; Babin et al. 2019; Hu et al. 2024), no prior study covering different biogeographical regions, to the best of our knowledge, compared the impacts of conventional and organic management on many different soil organisms. These new insights are essential for a good understanding of the potential biodiversity shifts in future transitions from conventional to organic farms. Moreover, the lack of an effect from shifts to organic farming on annelids and micro‐arthropods can be attributed to the simplicity of our dichotomy (conventional = chemical input, organic = no chemicals), as these organisms are known to be sensitive to soil management practices, such as no‐till (annelids) and buffer area inclusion (arthropods) (Cozim‐Melges et al. 2024).

Delving deeper into the taxonomy, different regional impacts related to soil management were also observed. For example, whereas a generally positive influence on potential plant growth‐promoting microorganisms was observed in organically managed soils (*Bacillus*, *Mycobacterium*, *Pseudonocardia*, *Streptomyces*, and *Trichoderma*), an increase in potential plant parasites, such as those from the fungal phylum Ascomycete (genus *Fusarium*) or the protistean phylum Gyrista (genus *Pythium*) (Blaya et al. 2013; Hamedi and Mohammadipanah 2015; Cui et al. 2019), was observed in soils under organic management from Belgium and Cameroon. Another example is the reduction of aerobic nitrifiers such as *Nitrospira* and methylotrophic bacteria such as *Hyphomicrobium* (Macey et al. 2020; Koch et al. 2019) and an increase in potential potassium‐solubilizing fungi such as *Mortierella* (Sang et al. 2022), specifically at organic sites in Latvia. Overall, the current results show the potential of organic soil management to shift microbial community dynamics towards plant growth promotion, but also towards the proliferation of potentially detrimental soil members, depending on the region where the soil is located. This is aligned with the lack of an overall consensus on how management practices affect the presence and dominance of pathogens (Larkin 2015). For instance, while some studies have shown that high organic matter inputs (e.g., manure) can suppress *Pythium* by enhancing microbial competition (Le et al. 2014; Mutai et al. 2024), the overall soil diversity reduction in Belgian organic soils in combination with a relative increase in *Pythium* among protists suggests that this region‐specific management may be lowering competition and allowing this potential pathogen to thrive. Moreover, the absence of chemical pesticides known to be effective against *Pythium* (Wu et al. 2020; Tsror et al. 2021) may have also contributed to the increased abundance of this genus. This highlights the need for continued improvement of biocontrol and pest monitoring strategies.

This study further assessed the potential dependence of soil management impacts on soil degradation. It is important to note that evaluating this relationship was particularly challenging in Belgium and Denmark due to the unbalanced representation of low‐degradation sites. Additionally, the mismatch in spatial resolution between erosion assessment (0.1–1 km^2^) and biodiversity sampling (1 m^2^) may have masked some effects of soil erosion on biodiversity. Otherwise, in reinforcement of the suggestion by Orgiazzi and Panagos (2018) for future research to better connect large‐scale erosion models with fine‐scale ecological responses, the present results show that the same soil management strategies can have different impacts on soil biodiversity depending on the soil degradation status and not only on biogeography. In Spanish soils, diversity benefits from organic management compared to conventional management were only observed in highly degraded soils. These results support the beneficial effects of soil restoration techniques that do not necessarily involve land‐use change, such as the implementation of organic farming strategies (Barral et al. 2015), which have also been proposed to mitigate water scarcity in arid regions (Sharma et al. 2025). In this line, the present results indicate that these restoration efforts may succeed, particularly in arid and highly degraded zones globally, to prevent potential biodiversity loss. However, the beneficial impacts will depend on the type of soil organism under assessment.

Based on this study, exploring the possibility of a transition towards organic agriculture at farm or regional levels by different types of stakeholders (landowners and policymakers), local and regional knowledge of climatic conditions, organic agricultural practices, and soil biodiversity will be crucial to protect and conserve soil organisms and the soil functions they drive. Particularly in the context of the Kunming–Montreal Global Biodiversity Framework and possible continental‐scale future policies, such as the Soil Monitoring Law in the EU, the results highlight that increasing knowledge at the regional scale is key to promoting effective soil management strategies and practices to conserve the different soil biodiversity components across agroecosystems.

## 5 Conclusions

Our study underscores the high regional specificity of soil communities with distinct compositions observed across continents and biogeographical regions. Soil prokaryotes and fungi were the most widespread taxa, with strong influence by climate and soil properties on their community composition determination. Although the global impact of soil management strategies was low compared with the other primary biodiversity drivers, certain soil organisms were particularly sensitive to the interactions among management, degradation, and climatic variables. Microbial taxa were more sensitive to environmental drivers and management practices than the fauna groups. Thus, the impacts of organic management on soil biodiversity are region‐ and organism‐specific. Benefits from organic management, in terms of sustaining soil biodiversity, were particularly pronounced in arid and highly degraded soils with low TOC and high pH. Future research on sustainable soil management in sensitive regions, particularly those similar to Mediterranean soils that are at high risk of degradation, is essential. In these regions, the interactions between degradation and management practices may have more pronounced effects than in soils with higher buffering capacities. Therefore, intensified efforts are needed to understand how soil management affects the full biodiversity spectrum and ecosystem functions in vulnerable environments, ultimately guiding effective conservation and restoration strategies.

## Author Contributions

**Pablo Sánchez‐Cueto:** data curation, formal analysis, investigation, methodology, visualization, writing – original draft, writing – review and editing. **Martin Hartmann:** conceptualization, funding acquisition, investigation, methodology, supervision, validation, writing – review and editing. **Laura García‐Velázquez:** data curation, formal analysis, investigation, methodology, writing – review and editing. **Beatriz Gozalo:** investigation, methodology. **Victoria Ochoa:** investigation, methodology. **Giulia Bongiorno:** investigation, methodology, writing – review and editing. **Ron Goede:** investigation, methodology, writing – review and editing. **Melpomeni Zoka:** conceptualization, data curation, formal analysis, investigation, methodology, writing – review and editing. **Nikolaos Stathopoulos:** data curation, formal analysis, investigation, methodology, writing – review and editing. **Charalampos Kontoes:** data curation, formal analysis, investigation, methodology, writing – review and editing. **Luis Daniel Olivares Martinez:** investigation, methodology, resources, writing – review and editing. **Jorge Mataix‐Solera:** investigation, methodology, resources, writing – review and editing. **Fuensanta García‐Orenes:** investigation, methodology, resources, writing – review and editing. **Tomas Van De Sande:** investigation, methodology, resources, writing – review and editing. **Helle Hestbjerg:** investigation, methodology, resources, writing – review and editing. **Ina Alsina:** investigation, methodology, resources, writing – review and editing. **Zoltán Tóth:** investigation, methodology, resources, writing – review and editing. **María Paula Barral:** investigation, methodology, resources, writing – review and editing. **Ximena Sirimarco:** investigation, methodology, resources, writing – review and editing. **Joseph Blaise Dongmo:** investigation, methodology, resources. **Julienne Nguefack:** investigation, methodology, resources. **Rochana Tangkoonboribun:** investigation, methodology, resources. **Anna Clocchiatti:** data curation, investigation, methodology. **Radu Ghemis:** supervision, writing – review and editing. **Montse Bosch:** funding acquisition, project administration, supervision, writing – review and editing. **Marcos Parras‐Moltó:** supervision, writing – review and editing. **Cristina Yacoub‐Lopez:** conceptualization, funding acquisition, project administration. **Santiago Soliveres:** conceptualization, data curation, investigation, methodology, supervision, validation, writing – review and editing. **Salvado Lladó:** conceptualization, funding acquisition, investigation, methodology, project administration, resources, supervision, validation, writing – review and editing.

## Conflicts of Interest

The authors declare no conflicts of interest.

## Supporting information


**Data S1:** gcb70486‐sup‐0001‐figures.docx.

**Data S2:** gcb70486‐sup‐0002‐Tables.xlsx.

## Data Availability

Sequencing data are available in the European Nucleotide Archive (ENA) at EMBL‐EBI under accession number PRJEB86495. Additional datasets supporting this study are openly available in Figshare at https://doi.org/10.6084/m9.figshare.30001381.
