# Supplementary material for: Impacts of Climate, Organic Management, and Degradation Status on Soil Biodiversity in Agroecosystems Worldwide
Source: Glob Chang Biol. 2025 Sep 12;31(9):e70486. doi: 10.1111/gcb.70486 (PMC12431722; doi:10.1111/gcb.70486)

Supplementary Figures

*Figure S1. Rarefaction curves by organism group*


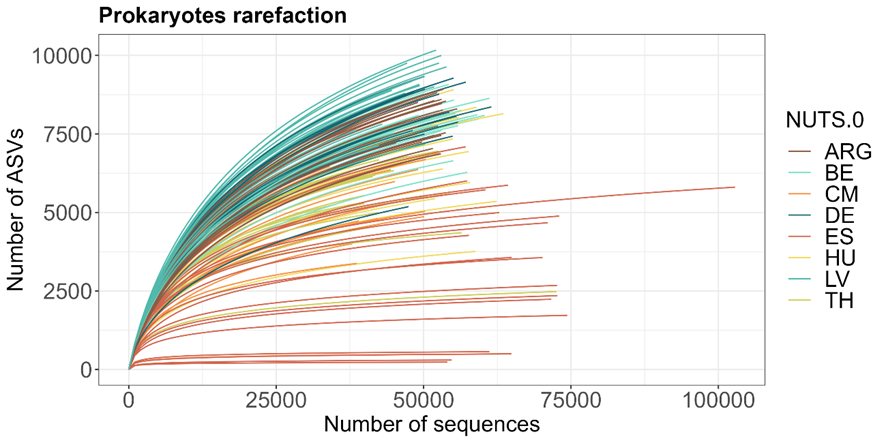

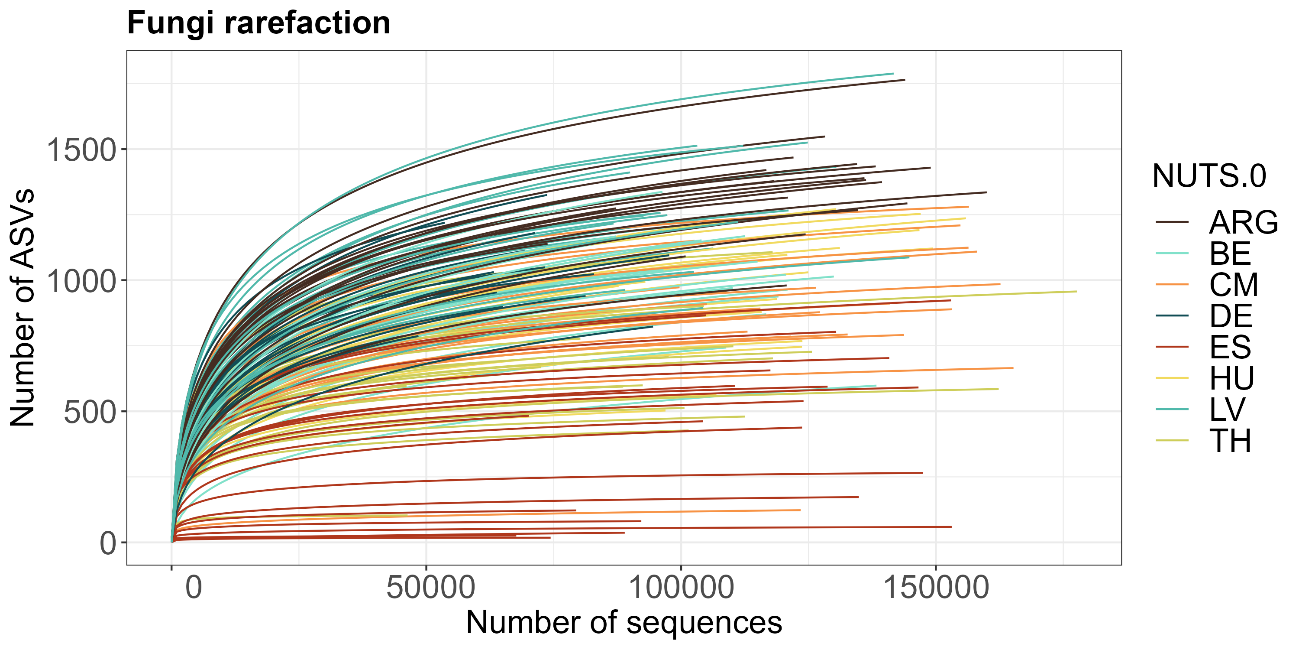


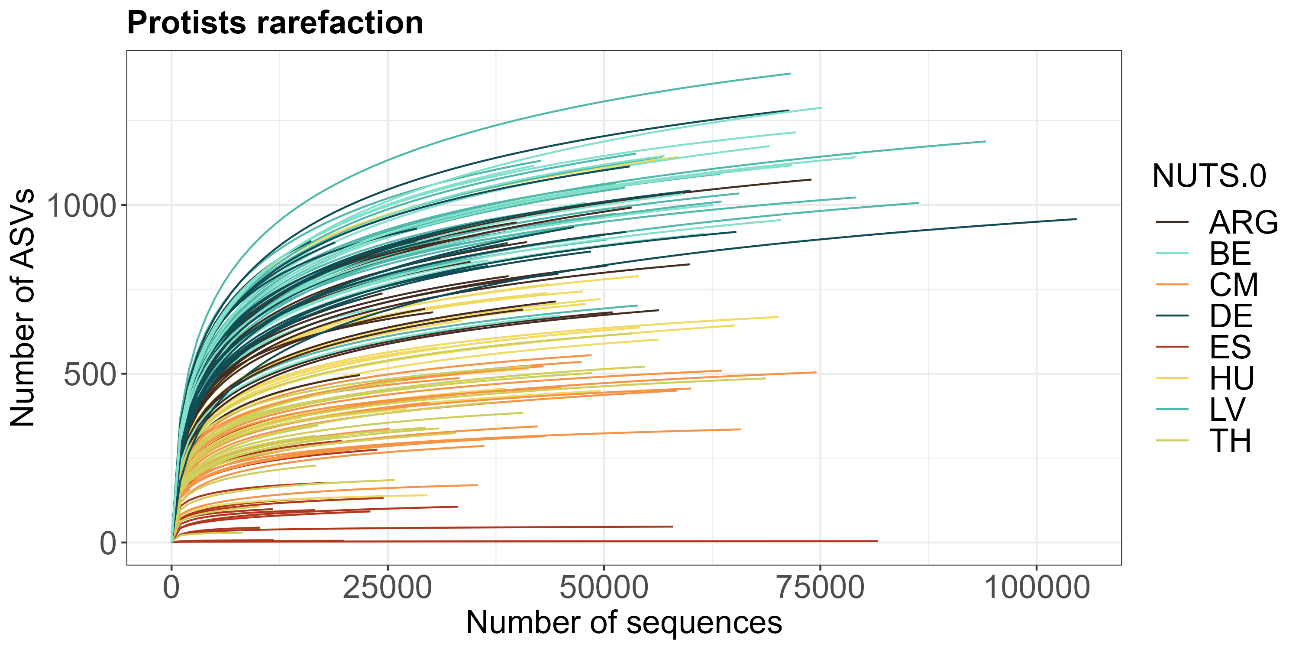

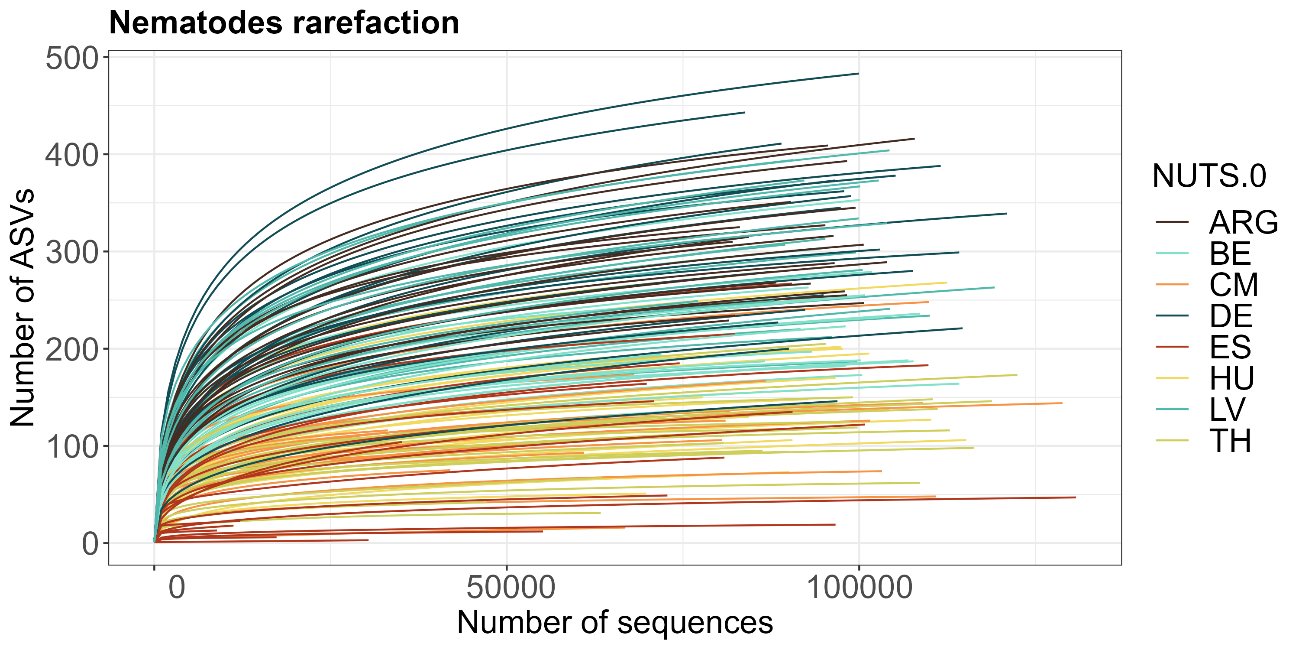


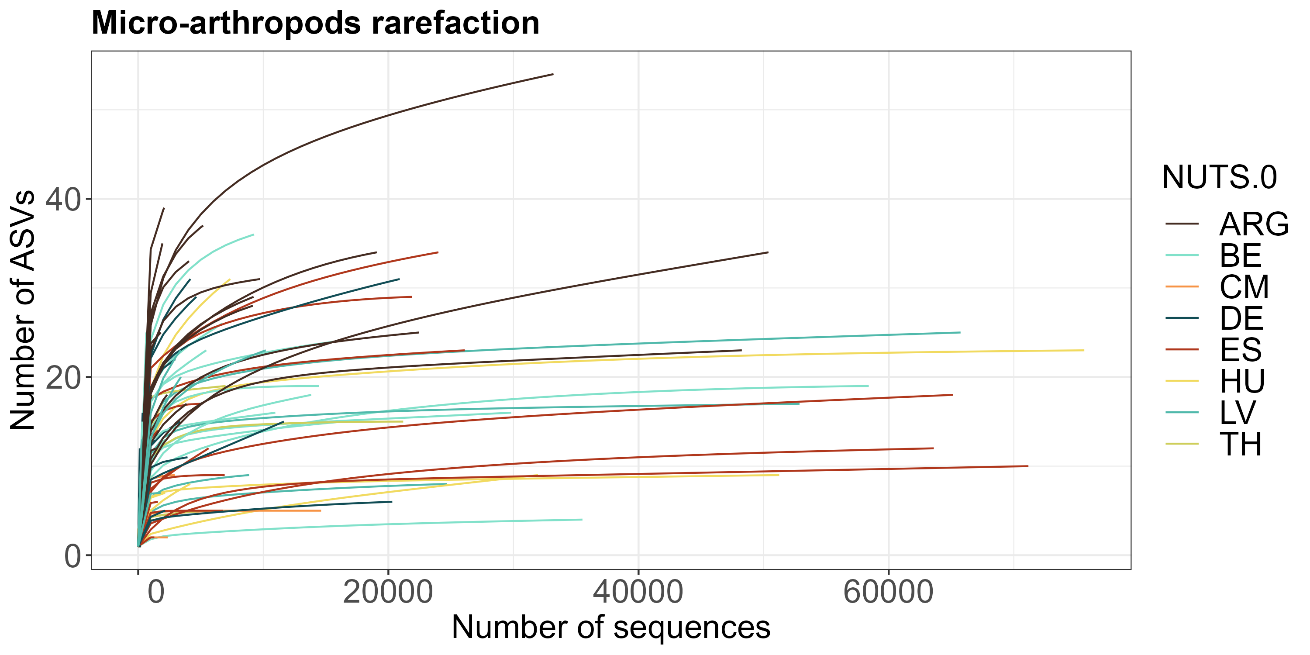


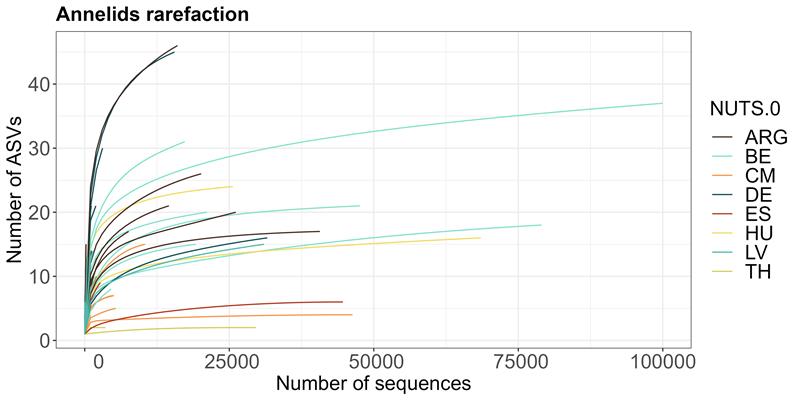


*Figure S2. Integrated overview of the present study. A) Distribution of the 160 croplands sampled. Both red and dark green points represent the location of conventional and organic agricultural management plots, respectively. These points are situated in areas with varying levels of soil degradation (low, medium, and high), indicated by co-assessing (within each country) soil erosion and soil organic carbon for each soil texture class. B) Laboratory assessment for soil biodviersity. Soil samples were collected from each plot and analyzed using DNA-based molecular techniques targeting key soil organisms. Specific genetic markers were amplified and sequenced to characterize the diversity of prokaryotes, fungi, protists, and metazoans (Table S1E). C) Statistical analysis framework. Multiple biodiversity metrics were computed from the molecular data, including Amplicon Sequences Variants (ASVs) relative abundance, alpha diversity and beta diversity. Data was processed to i) assess the global status of biodiversity, ii) global and iii) regional main drivers and iv) sensitive taxa to management. Part of main figures associated to each objective are shown in their respective boxes.*


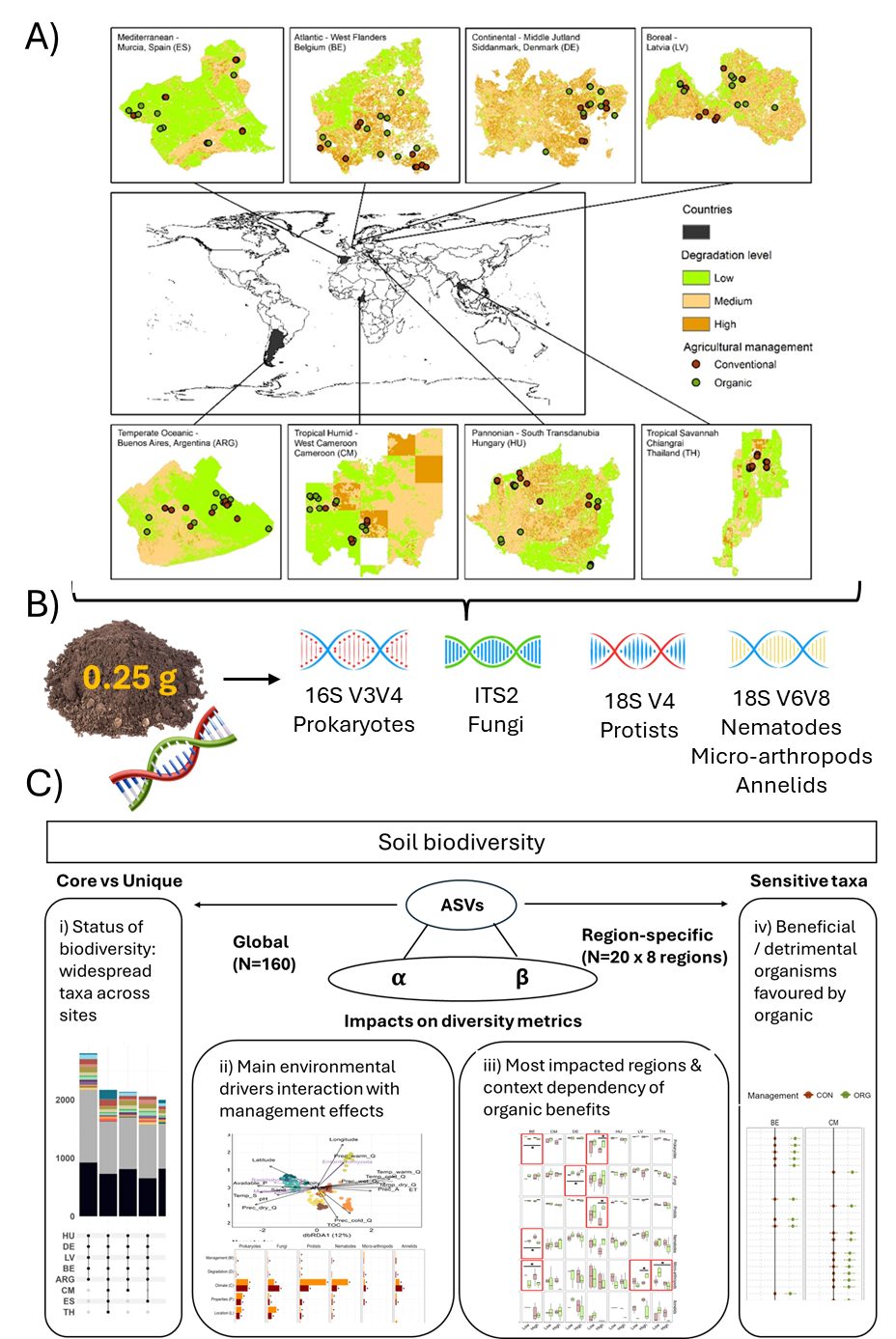


*Figure S3. Most abundant phyla for microbial organisms ((a) prokaryotes, (b) fungi and (c) protist) or order for metazoan ((d) nematodes, (e) micro-arthropods and (f) annelids), by region, management and soil degradation level.*

1. Prokaryotes


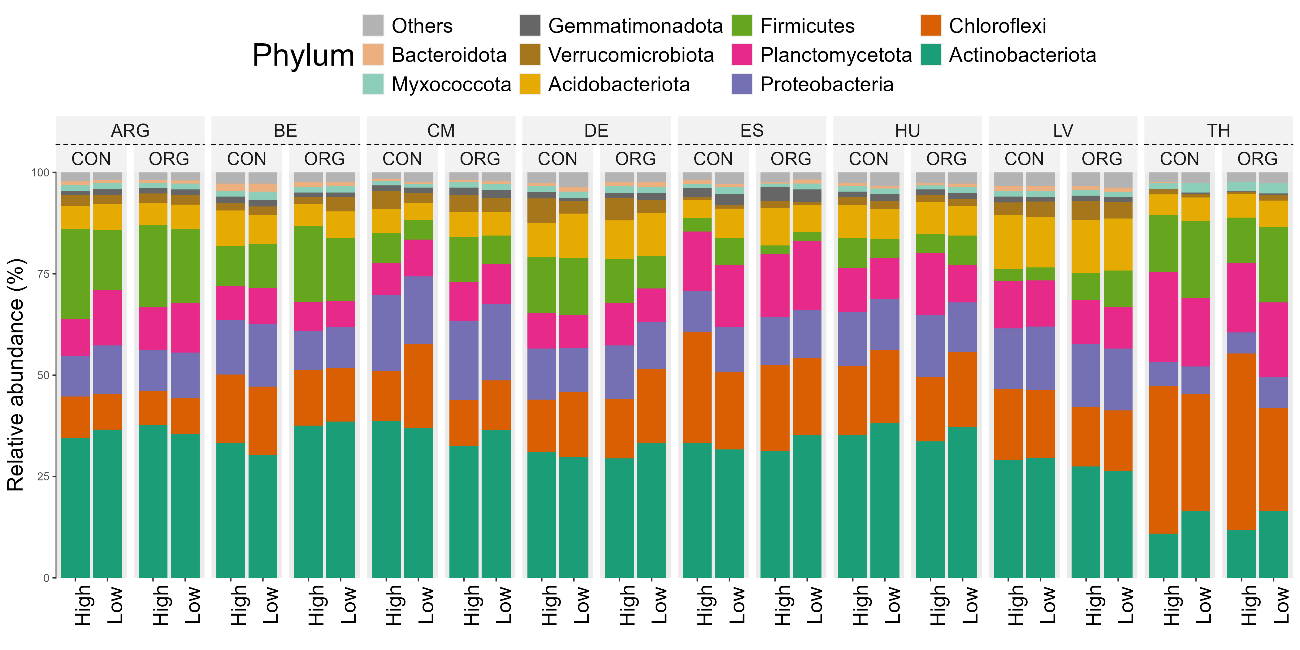


1. Fungi


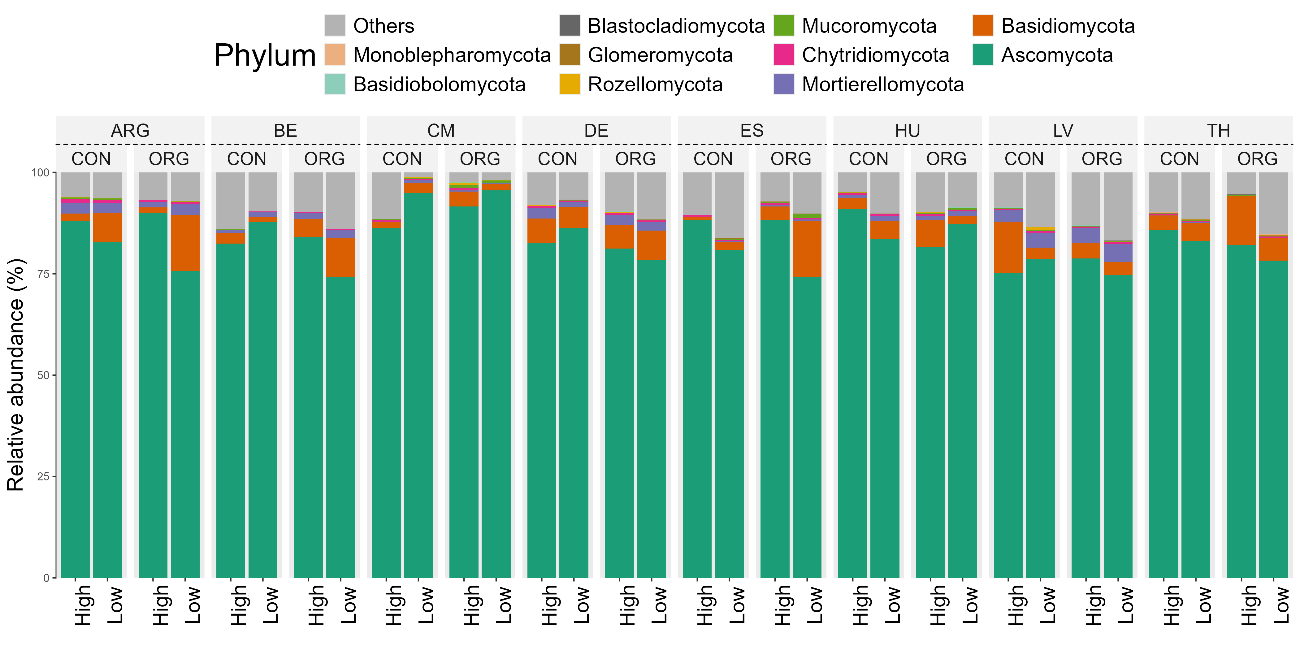


1. Protists


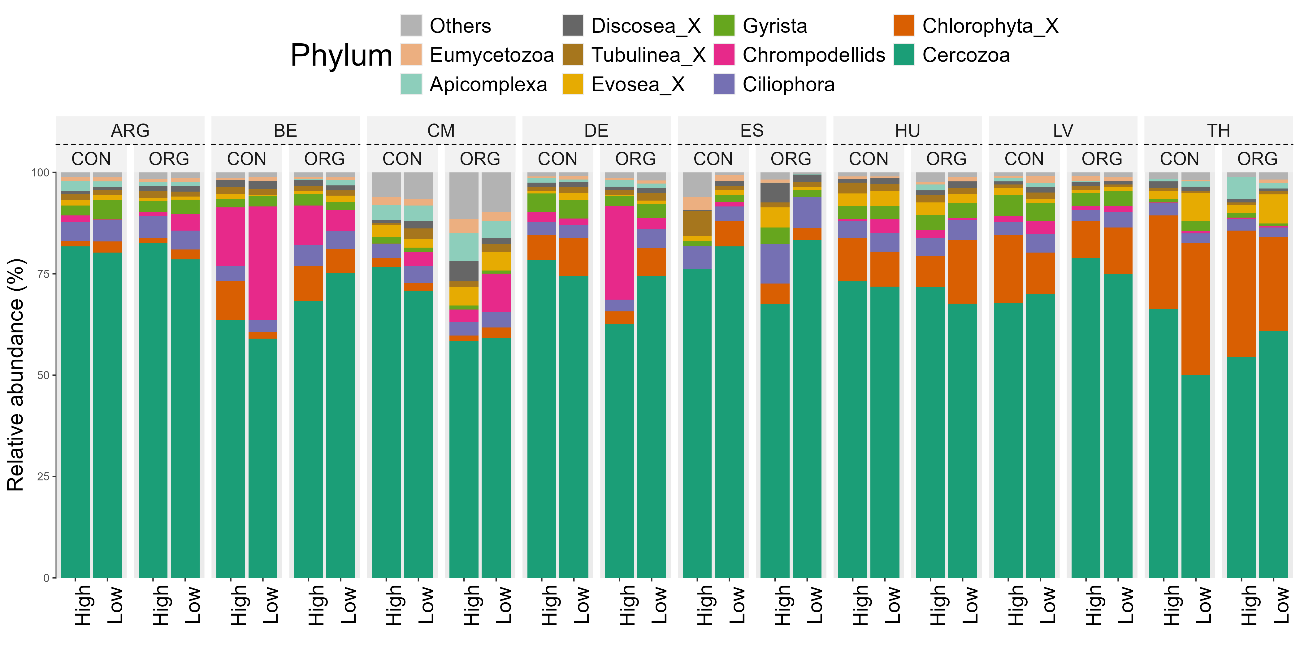


1. Nematodes


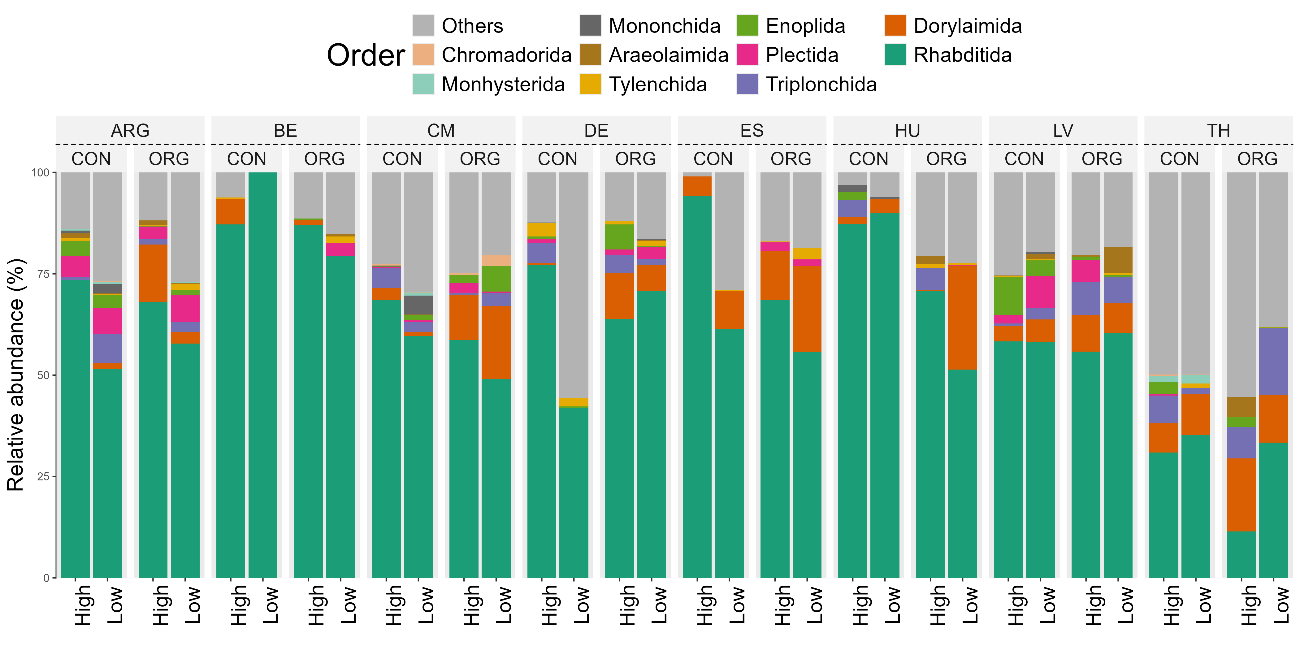


1. Micro-arthropods


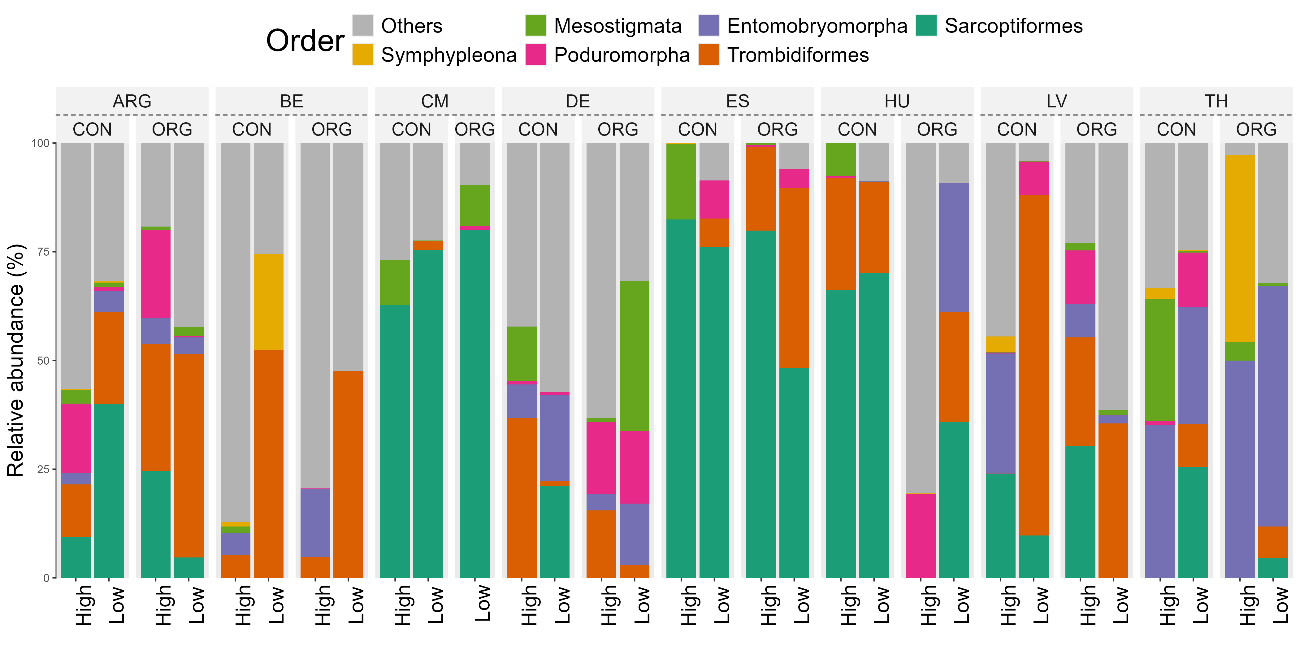


1. Annelids


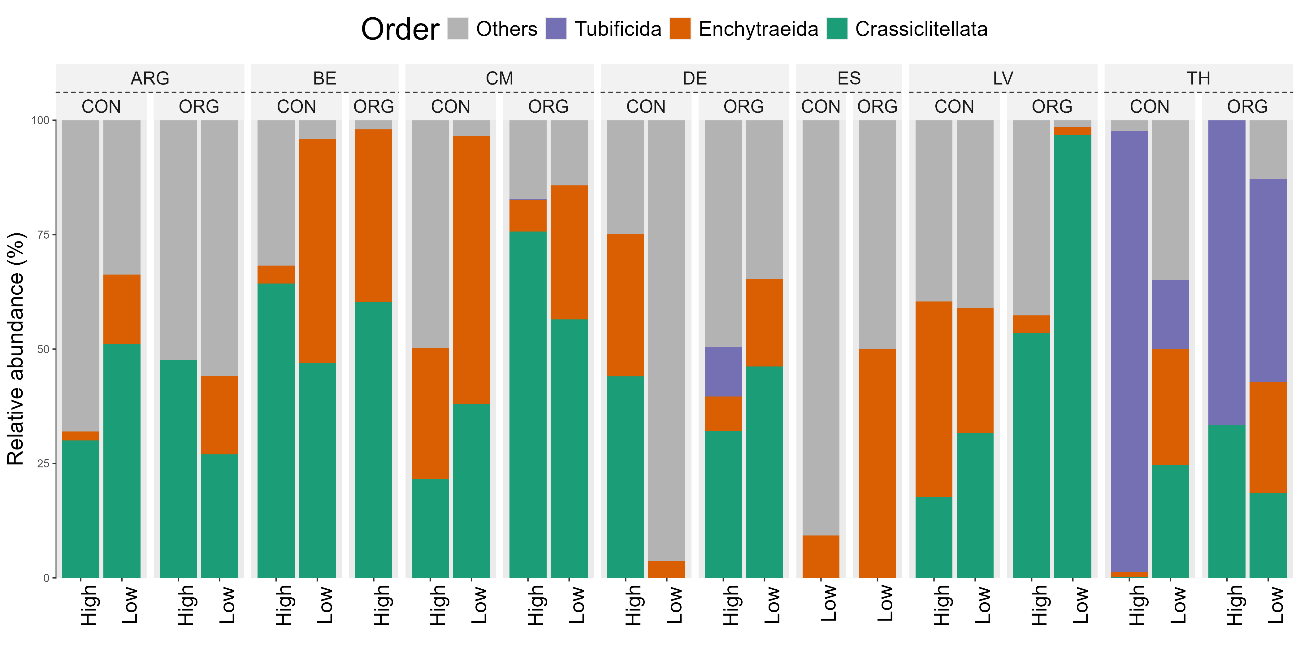


*Figure S4. Distribution of shared ASVs between regions. (a) Proportion (bars) and cumulative relative abundance (dots) of shared vs. unique ASVs across regions, by target organism. Two-axis plot shows the distribution of "core" and "unique" ASVs across the 8 regions, grouped by target organism. The first axis (left) represents with bars the proportion of shared versus unique ASVs between regions, where "core" ASVs are those shared across all regions, and "unique" ASVs are those found in only one region. The second axis (right) displays with dots the mean cumulative relative abundance of these ASVs per region, indicating how dominant the shared and unique ASVs are across the regions. (b)* ***I****ntersection bar plot of shared ASVs across regions, grouped by taxa and genus level. The barplots represent the size (number) of the shared of ASVs, further classified at the genus level, providing a more detailed view of the taxa composition across regions. Each dot below in the intersection indicates that these ASV are shared between the specified regions. Region name is abbreviated and color coded (only in S4a) by the country of origin: ARG-Argentina, DE-Denmark, HU-Hungary-, LV-Latvia, Be-Belgium, ES-Spain, TH-Thailand, CM-Cameroon.*

*
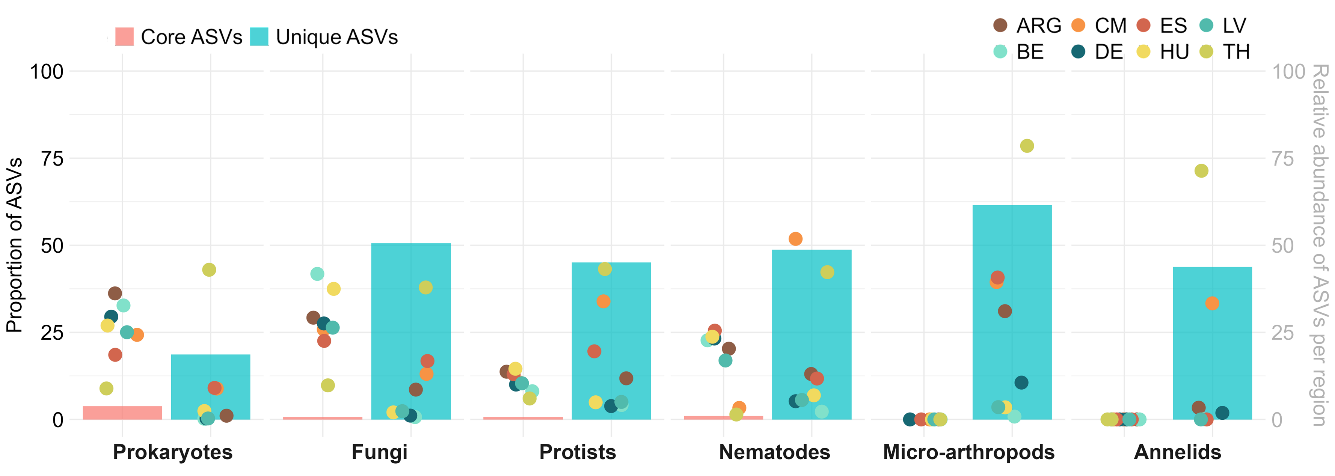
*

(a)

***
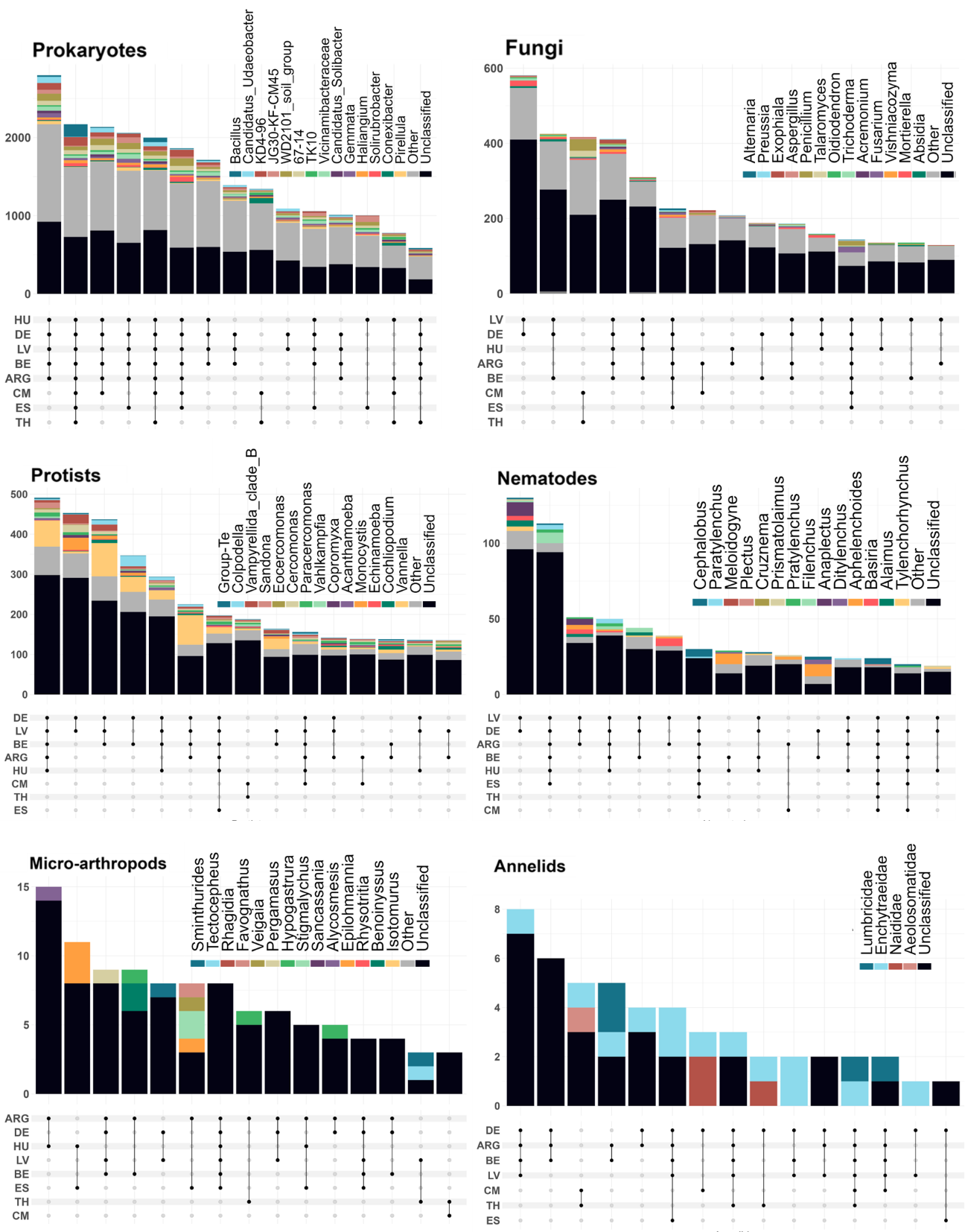
***

(b)

Figure S5. *dbRDA analysis, by organism group, on Bray-Curtis dissimilarity matrix calculated from ASV counts, showing the influence of soil physicochemical properties, climatic variables and location (in black); and their relationship with Phylum and order significatively associated (rho > 0.5; p < 0.05) with the sample’s ordination are shown in purple. Region name is abbreviated and color coded by the country of origin: ARG-Argentina, DE-Denmark, HU-Hungary, LV-Latvia, Be-Belgium, ES-Spain, TH-Thailand, CM-Cameroon.*

*
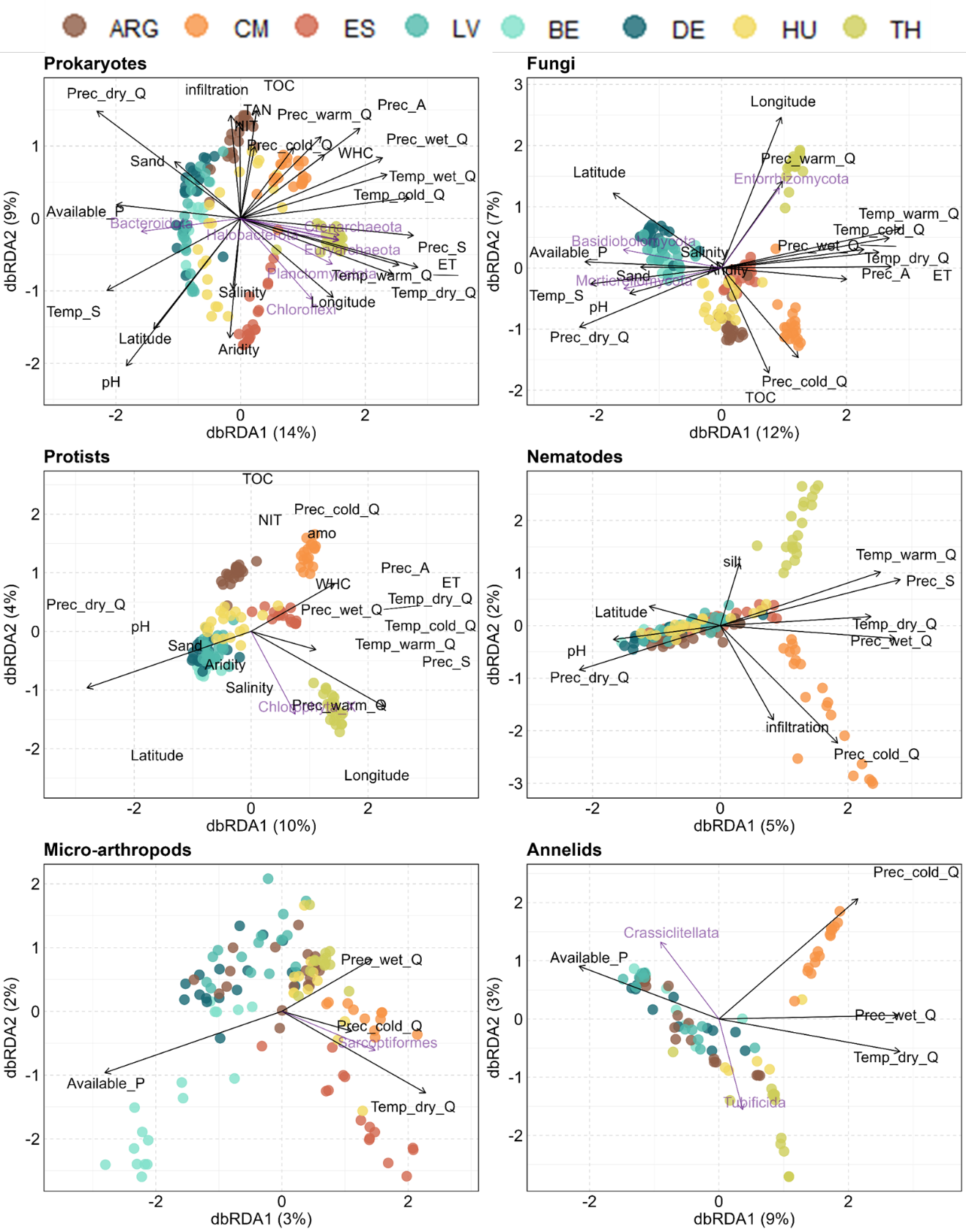
*

Figure S6. *dbRDA analysis of prokaryotes, fungi and protists, on Bray-Curtis dissimilarity matrix calculated from ASV counts, showing the influence of soil physicochemical properties, climatic variables and location (in black); and their relationship with orders relative abundance (significatively associated (rho > 0.3 and p < 0.05), in purple). Region name is abbreviated and color coded by the country of origin: ARG-Argentina, DE-Denmark, HU-Hungary, LV-Latvia, Be-Belgium, ES-Spain, TH-Thailand, CM-Cameroon.*

*
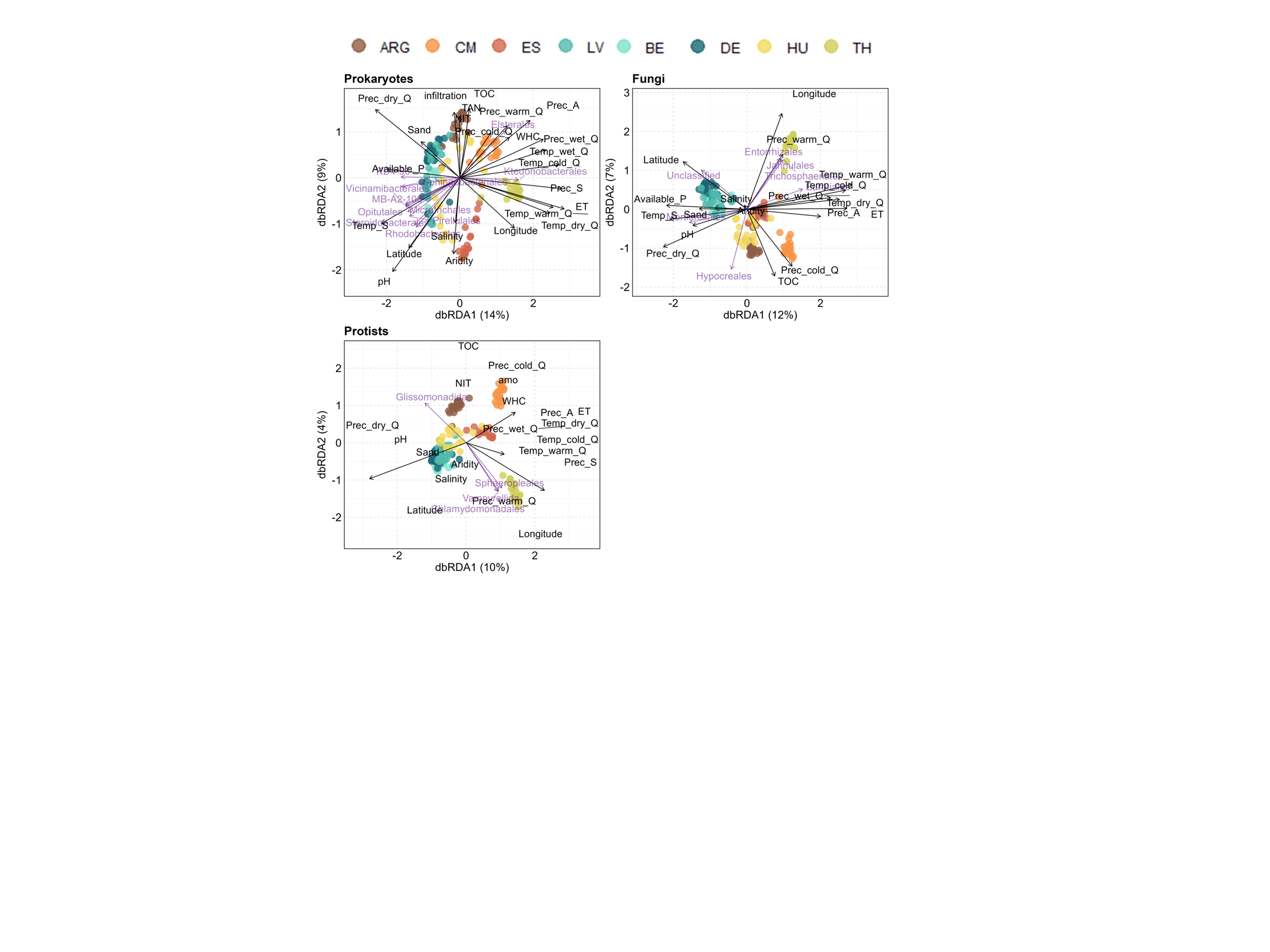
*

Figure S7. *dbRDA analysis, by organism group, on Bray-Curtis dissimilarity matrix calculated from ASV counts, showing the influence of soil physicochemical properties, climatic variables and location (in black); and their relationship with genera relative abundance (significatively associated (rho > 0.3 and p < 0.05, in purple). Region name is abbreviated and color coded by the country of origin: ARG-Argentina, DE-Denmark, HU-Hungary, LV-Latvia, Be-Belgium, ES-Spain, TH-Thailand, CM-Cameroon.*

*
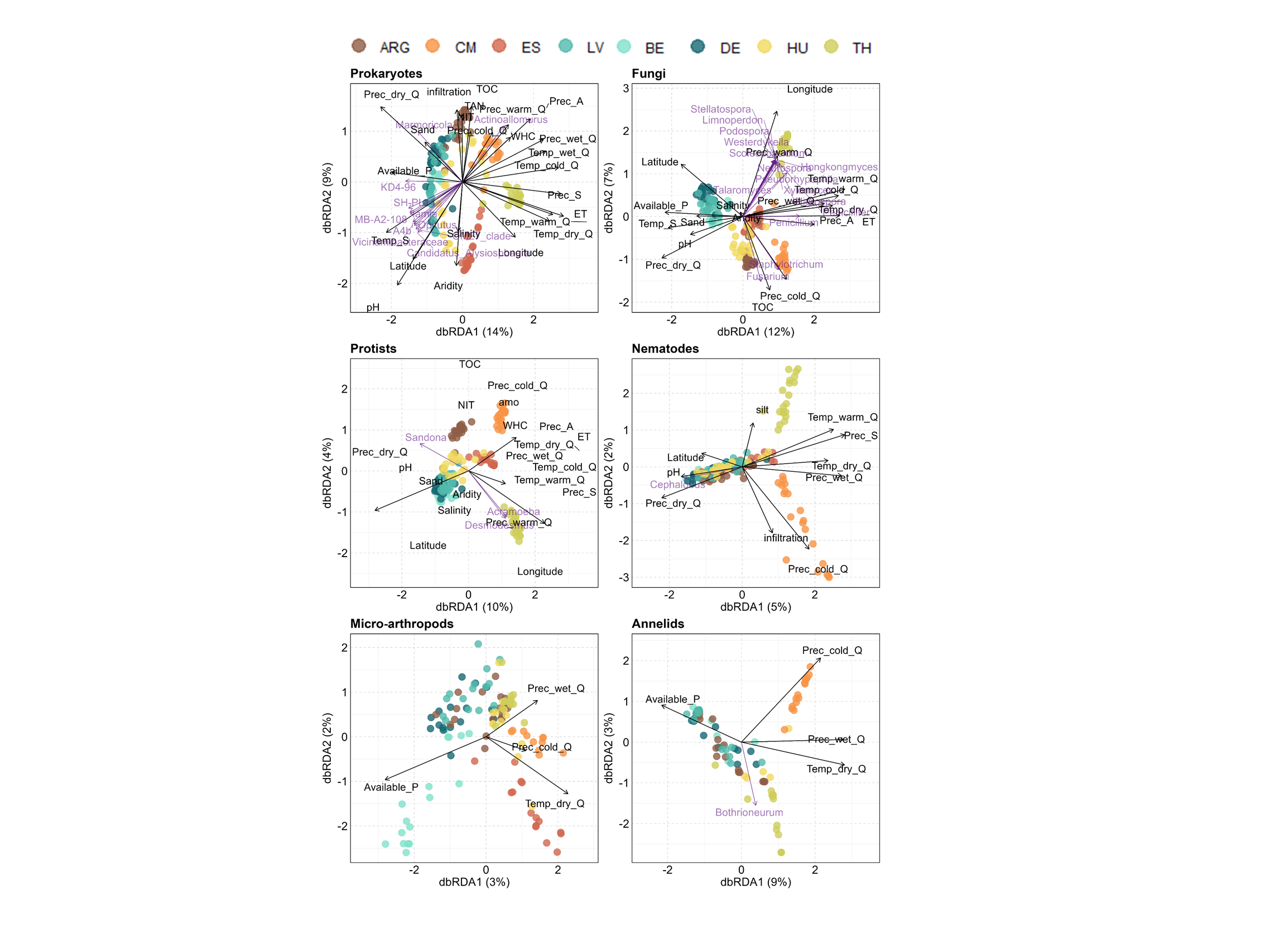
*

*Figure S8. Intersection bar plot showing the number and distribution of shared ASVs across different combinations of management type (conventional vs. organic) and degradation level (low, high) in Spanish (Murcia) soils. This region is highlighted because it showed the strongest combined effect of management and degradation on alpha diversity (see Table S8). The plot illustrates the number of ASVs that are unique or common across management–degradation categories.*


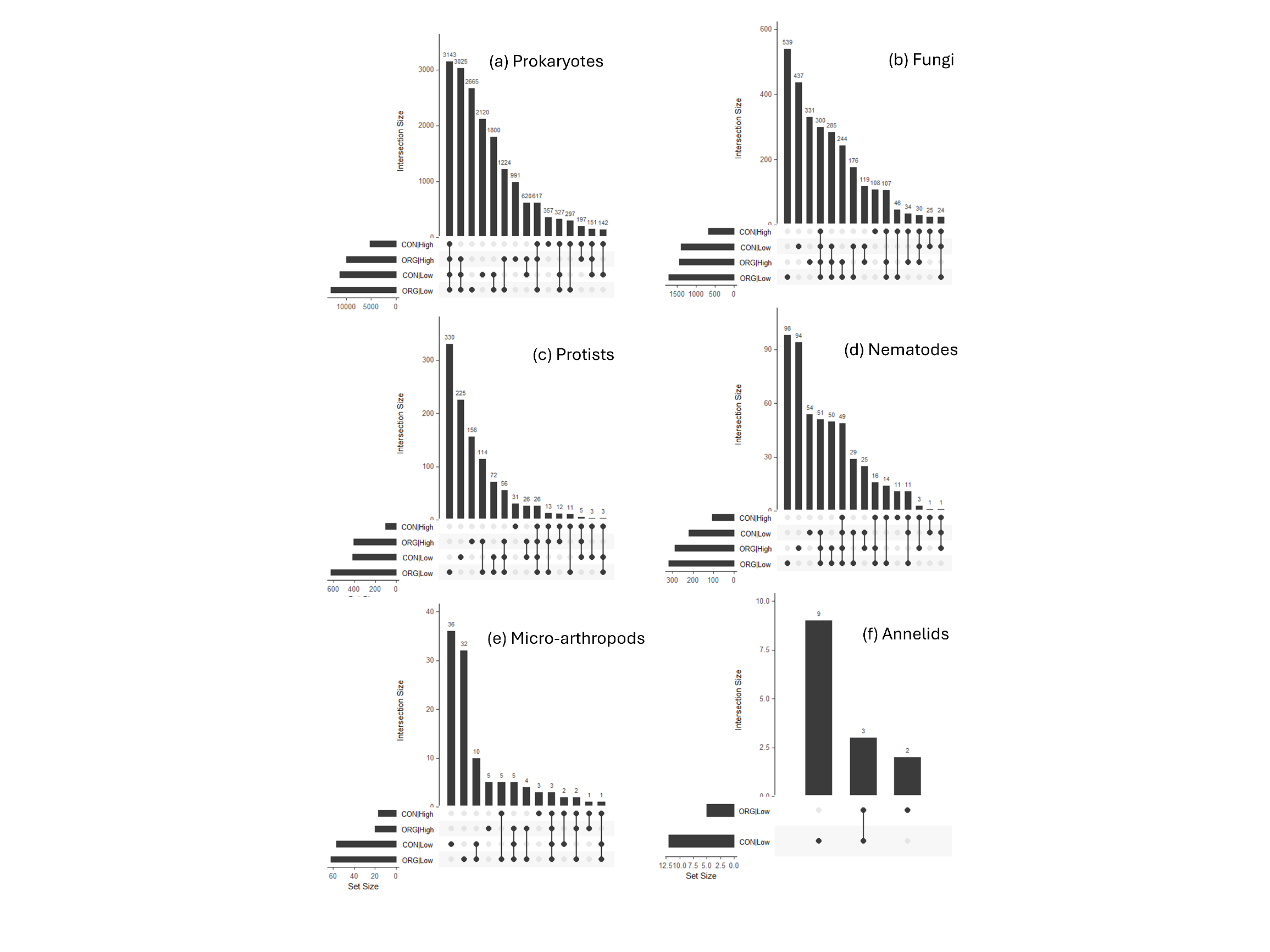

Supplement: Supplementary file 1 — Data S1: gcb70486‐sup‐0001‐figures.docx. [file GCB-31-e70486-s001.docx]
